# Staphyloxanthin loaded niosomal nanocarrier augments its anthelmintic activity against *Trichinella spiralis* infection in mice

**DOI:** 10.1038/s41598-025-17936-9

**Published:** 2025-09-12

**Authors:** Ahmed M. Nosair, Ahmed A. Abdelaziz, Amal M. Abo-Kamar, Hager S. Zoghroban, Mahmoud H. Farghali, Lamiaa A. Al-Madboly

**Affiliations:** 1 Department of Microbiology and Immunology, Faculty of Pharmacy, Tanta University, Tanta, 31527 Egypt; 2 Department of Medical Parasitology, Faculty of Medicine, Tanta University, Tanta, 31527 Egypt

**Keywords:** Trichinellosis, Staphyloxanthin, Nanocarrier, Niosomes, Target proteins, Biological techniques, Drug discovery, Immunology, Microbiology, Molecular biology, Zoology

## Abstract

Trichinellosis, a resurgent zoonotic infestation, threatens public health due to recorded human outbreaks in various nations. The emergence of treatment resistance necessitates the exploration of efficient natural alternatives. Staphyloxanthin (STX), a membrane-associated secondary metabolite carotenoid pigment, underscores pro-oxidative traits, positioning it as a novel therapeutic candidate. Nanostructures demonstrated encouraging promise in overcoming low oral bioavailability, which could undermine the efficacy. Hence, the therapeutic outcome of STX-loaded niosomes was scrutinized both in vitro and in vivo. In this study, the prepared niosomal nanovesicles exhibited a spherical form in the nanoscale spectrum. Our in vitro findings demonstrated that STX markedly diminished larval viability, associated with excessive cuticular deformities, numerous notches, and membrane blebbing. The preclinical evaluation revealed that the oral delivery of STX-niosomes showed a superiority of therapeutic efficacy in mice compared to the reference drug. This was reflected by the eradicated adult worms, enhanced histopathological attributes, and reduced larval count. It is noteworthy that the biological findings revealed a significant reduction in the inflammatory expression of TNF-α surrounding trichina capsules. The relationship between STX and the parasite was elucidated, with the promising antiparasitic efficacy being further corroborated through in silico homology modelling and molecular docking approaches. The 3D-modelled target protein structures exhibited excellent quality factors and favourable Ramachandran plot statistics. Intriguingly, in silico docking results obviously revealed the potential affinity of STX to bind and block target protein receptors. In conclusion, our results suggested that STX pigment may serve as a promising pioneering alternative in the anthelmintic fight against trichinellosis.

**Supplementary Information:**

The online version contains supplementary material available at 10.1038/s41598-025-17936-9.

## Introduction

*Trichinella spiralis* is a parasitic nematode associated with food-borne disease, known as trichinellosis, which is frequently acquired through the consumption of undercooked meat, predominantly pork and its derived goods harboring the encysted larvae^1^. Due to its diverse host range, encompassing 150 distinct species of animals as well as humans, trichinellosis presents an urgent public health challenge; with an estimated 11 million individuals infected worldwide^2^. Out of 24 parasites associated with international trade, *T. spiralis* has been deemed the most noteworthy by the Food and Agriculture Organization (FAO) of the United Nations (UN) and the World Health Organization (WHO)^3,4^. *T. spiralis* undergoes three distinct stages in its life cycle: the adult form, newborn larvae (NBL), and infectious muscle larvae (ML)^5^. Throughout these phases, cysteine proteases are released, serving a pivotal function in pathogenesis by aiding in metabolic processes related to nutrition, molting, tissue infiltration, evasion of immune responses, and/or alterations^6^. An interesting finding about a cysteine protease from *T. spiralis* (Ts-CF1) is its expression throughout life cycles, as well as its localization within the stichosome and cuticle. This finding lends credence to the idea that Ts-CF1 could be a prospective therapeutic target or vaccine candidate for *T. spiralis* infection^7,8^. Moreover, this parasite exhibits a diverse array of strategies for immune evasion, skillfully circumventing both the host’s adaptive and innate defense mechanisms^9^. Calreticulin (Ts-CRT) represents a sophisticated mechanism that enables survival within a challenging host environment by engaging in overlapping interactions, and disrupting the complement immune system within the host^10^.

The parasite’s β-tubulin monomer is selectively bound to the benzimidazole-2-carbamate derivatives (BzC) as the approved treatment, thereby inhibiting the polymerization of microtubules^11^. Nevertheless, the efficacy of the treatment is significantly diminished owing to the mammalian host’s tubulin binding. Although they are not recommended during pregnancy and discouraged for children under the age of two, they are also not entirely effective against newborn or encysted larvae due to the emergence of drug resistance and low bioavailability^11^. The ongoing evolution of helminth resistance to synthetic anthelmintic medications, however, calls for the investigation of natural compounds as a potential replacement for these drugs^12^. On top of that, natural pigments tend to be more accessible and economically feasible^13^.

A multitude of studies has recently explored the potential uses of natural pigments, such as carotenoids, in the fight against both non-parasitic and parasitic infections. The findings of these studies have been shaped by their ethnopharmacological relevance in addressing coughs respiratory ailments, dysentery, diarrhea, gastrointestinal parasite infections, and rheumatic diseases^14–16^. Staphyloxanthin (STX) is a notable pigment derived from *Staphylococcus aureus*, classified within a distinct category of apocarotenoid triterpenoid pigments^17^. In microorganisms and plants, oxidative cleavage of C40 isoprenoids produces apocarotenoids. Carotenoid cleavage dioxygenases (CCDs) play a major role in the enzymatic production of apocarotenoids, whereas additional processes are used in the non-enzymatic process^17^. The biological potential of carotenoids is chiefly ascribed to the production of reactive oxygen species (ROS), leading to oxidative stress and suppression of ion-membrane interactions and active solute transport, thereby altering the cellular permeability^18^. Regarding to the aforementioned scenario, this study relies on the evaluation of STX anthelmintic activity against *T. spiralis*.

The intrinsic physicochemical characteristics, such as low solubility in water and insufficient permeability, present a significant challenge and restrict the oral administration of certain drugs due to their suboptimal bioavailability^19^. Nano-carriers such as niosomes have been widely employed to facilitate drug delivery and enhance their oral bioavailability. Niosomes are bilayered structures consisting of biocompatible materials, specifically non-ionic surfactants and cholesterol, which serve as lipids^20^. These nanovesicles are water-soluble, exhibiting exceptional biocompatibility and possessing the capability to carry both hydrophobic and hydrophilic agents. This approach of drug delivery significantly improves the physical stability of the pharmaceutical compound, preventing chemical deterioration and the detrimental effects of unfavorable environmental conditions^21^. Therefore, STX was formulated as a niosomal dispersion to improve oral bioavailability and enhance the therapeutic efficacy against *T. spiralis*.

To the best of our knowledge, this is the first report to examine the impact of STX on the treatment of trichinellosis. The objective of this research was to assess the novel antiparasitic activity of STX against *T. spiralis* both in vitro and in vivo as a drug solution and niosomal dispersion. Moreover, homology modeling and molecular docking analysis were performed to investigate the potential affinity of STX to block *T. spiralis*-target proteins.

## Materials and methods

### Materials

The STX pigment was extracted from *Staphylococcus aureus* A2 (accession number PP197164). The laboratory extraction, purification, chemical validation, and safety profile on normal Vero cells of STX were covered in our previous research^22^. Cholesterol and sorbitan monostearate (Span 60) (Sigma Chemical Co., St. Louis, MO, USA) were obtained from the Pharmaceutical Technology Department, Faculty of Pharmacy, Tanta University, Egypt. Albendazole (ABZ) served as the reference anthelmintic drug, supplied by Pharma Cure Pharmaceutical Industries, Egypt, as a 400 mg/10 mL oral suspension (Alzental).

### In silico pharmacokinetics, drug‑likeness, and toxicity predictions

STX and ABZ were transformed into their canonical simplified molecular-input line-entry system (SMILE) representations. Subsequently, these models were analyzed using the ADMETlab 3.0 server to determine their pharmacokinetic parameters. This included assessments of **A** (absorption, specifically MDCK, skin permeability, human intestinal absorption (HIA), and Caco-2), **D** (distribution, encompassing Blood-Brain barrier penetration (BBB), P-glycoprotein substrate, inhibitor, and plasma protein binding (PPB)), **M** (metabolism, focusing on CYP family substrate/inhibitor), **E** (excretion, particularly elimination half-life (t_1/2_)), and **T** (toxicity, including carcinogenesis and ames mutagenesis), in addition to the evaluation of the drug-likeness score.

### Preparation of Staphyloxanthin-loaded Niosomal formulation

The composition of the niosomal formulation listed in Table 1 was utilized following the previously reported investigation^23^. Niosomes were formulated using hydration method as previously described^21,23^. In brief, span 60 (surfactant) and cholesterol were melted on a water bath at 55 °C. STX was dissolved in 5 mL ethanol and the solution was then added to the melted components with mixing to form clear liquid. A homogeneous mixture was formed by gradually incorporating 5 mL of water upon mixing. Proniosomal creamy gel was formed with continuous mixing upon cooling. Hydration of proniosomes was used to prepare the surfactant nanovesicles. This was accomplished through the incremental incorporation of the remaining water, with continuous mixing to develop homogenous niosomal dispersion. The niosomal dispersion was allowed to undergo hydration and swelling by overnight incubation at room temperate. For size reduction, the prepared swollen vesicles were bath sonicated for 45 min. Ice cubes were used to prevent heating of the sample during sonication. This technique produced STX-loaded niosome at a final concentation of 12 mg/mL.

**Table 1:** Composition of Niosomal formulation.

Formulation	Amount or volume
Span 60	1.2 g
Cholesterol	0.3 g
Ethanol	3 mL
Staphyloxanthin	0.3 g
Distilled water	Up to 25 mL

### Morphology and vesicle size determination

The morphological attributes and size of the prepared niosomal formulation were examined utilizing a transmission electron microscope (TEM) (JEOL – JSM1400 PLUS, Tokyo, Japan)^21^. Niosomes were suitably diluted (1:200) with HPLC filtered distilled water. One droplet of niosomal dispersion was loaded on a carbon grid and left to dry. Afterwards, the grid endured a sequential staining process, first with saturated uranyl acetate dissolved in 70% ethanol for 5 min, followed by lead citrate for 2 min. The sample was delicately affixed in the holder for examination under the electron microscope. Photomicrographs were obtained through meticulous calibration of the magnification power.

### Dynamic light scattering spectroscopy (DLS)

The determination of niosome size was further validated using DLS, which yields the average vesicle size (Z-average)^24^. This analysis was conducted utilizing the Zetasizer (Zetasizer Nano ZS, Malvern Instruments Ltd, Malvern, Worcestershire, United Kingdom). The uniformty of vesicle dispersion were also assessed using DLS, as indicated by the polydispersity index (PDI) value. The same technique was employed to record the zeta potential. HPLC filtered distilled water was used to appropriately dilute (1:200) the formulation before loading into the cell. The measurements were taken with the diffraction angle set to 90° at 25 °C. The mean of 14 measurements was used to calculate the Z-average. The system software was used to calculate the PDI value. The value of zeta potential was ascertained from a comprehensive dataset comprising 100 determinations.

### Entrapment efficiency and drug loading

The evaluation of the encapsulation efficiency of the niosomes was conducted after the separation of the free STX pigment, as previously described^20,25^. This was accomplished by centrifuging the prepared nanovesicles for 90 min at 12,000x g, and then carefully separating the liquid phase supernatant from the solid residue pellet. The unentrapped STX content in supernatant was determined spectrophotometrically by measuring the absorbance at 456 nm. The entrapment efficiency EE% was calculated by subtracting the free STX content from the total amount of STX initially used in vesical formulation, according to the provided equation:

$$\text{EE}{\%}=[(\text{A-B)/A}]\times100$$

A: The total amount of STX initially incorporated in vesical formulation. B: The unentrapped STX content in supernatant.

The loading efficiency (LE) was shown as the amount of STX encapsulated per lipid. This gave a more accurate picture of the niosomes’ ability to load STX compared to the total amount of lipid in the formulation, as shown in the equation:

LE (mg/g) = actual amount of encapsulated drug/total lipid in the formulation.

### Stability of niosomes

The stability of niosomes can be confirmed by measuring particular characteristics over a specified period^25^. To put it briefly, 1 mL of STX-nisomes’ size and EE% were evaluated. For a month, the requirements were observed at two distinct temperatures: 4 and 25 °C.

## Evaluation of anthelmintic activity

### Parasite and animals

Male Swiss albino mice used in the study were parasite-free, laboratory-bred, and weighed an average of 25 g. The mice were purchased from the animal house at the College of Veterinary Medicine of Cairo University (Cairo, Egypt). The ethics committee of Faculty of Pharmacy, Tanta University approved the care and manipulation of the animals, which were carried out in accordance with the National Institutes of Health’s guide for the use and care of laboratory animals (TP/RE/3/25 p-01). Mice were fed a standard pellet diet and had unrestricted access to water. The *T. spiralis* (code: ISS6158) utilized in this investigation was supplied by the Medical Parasitology Department, Faculty of Medicine, Tanta University, Egypt. The initial isolate of *T. spiralis* was procured from infected pork sourced from a slaughterhouse in Cairo. This was upheld through continuous transmission to mice and rats. This *Trichinella* isolate was genotyped as *T. spiralis* at the Superior Institute of Health in Rome, Italy, which is the European Union Reference Laboratory for Parasites. Each mouse was infected with 200–300 larvae of *T. spiralis* through oral administration^26,27^.

### Isolation of *T. spiralis* adult worms and muscle larvae

Muscle larvae and adults of *T. spiralis* were collected from the infected animals, as previously described^27^. After 30 days of infection with *T. spiralis*, the Swiss albino mice were sacrificed, their muscles were removed and ground, and the larvae of the infected muscles were digested by submerging them in a solution of acid pepsin. The mixture was stirred continuously for 2 h at 37 °C before the digest was filtered. The recovered larvae were rinsed with tap water many times and then let to settle in a conical flask for 30 min. In order to isolate adult *T. spiralis* worms, the small intestines of infected untreated mice were washed and then cut longitudinally along their entire length. The resulting pieces were 2 cm in diameter, and they were then placed in normal saline at 37 °C for 3 or 4 h to facilitate the worms’ migration out of the tissue.

### In vitro experimental design

A sterile 24-well tissue culture plate from SoCal BioMed in the USA was used for all investigations. A 2 ml of RPMI-1640 medium (Lonza, Belgium) supplemented with 10% fetal bovine serum, 200 µg/ml streptomycin, and 200 U/ml penicillin (Omega Scientifc, USA) was used to incubate muscle larvae (ML) of *T. spiralis* (60 ML per well). This study established five distinct groups as follows: Group I consists of muscle larvae cultured solely in RPMI, while Group II comprises muscle larvae cultured in RPMI that includes purified STX pigment, dissolved in 1% dimethyl sulfoxide (DMSO) at a concentration of 100 µg/mL. Group III: muscle larvae cultured in RPMI with ABZ at a concentration of 100 µg/mL. Group IV: muscle larvae cultured in RPMI with the niosomal formulation of STX at a concentration of 100 µg/mL. Group V: muscle larvae cultivated in an incubation medium comprising a combination of STX niosomes (50 µg/mL) and ABZ (50 µg/mL). The experiment was conducted in four replicates. The plates were meticulously sealed and incubated at a controlled temperature of 37 °C within an environment enriched with 5% CO_2_ for durations of 1, 6, 24, and 48 h^28^. The assessment of parasite viability was conducted through manual counting. To evaluate the viability rates of parasites, only definitively dead parasites were counted. Despite exhibiting minimal motility, the parasites were classified as living organisms. The survival rate in each well was determined by considering that non-motile worms were deceased. The criteria for a deceased body were as follows: the parasite’s body was either linear, comma-shaped, or immotile. Parasite mortality % =(the number of dead ML/the total number of ML)×100%^27^.

### Ultrastructural examination using scanning electron microscopy

The effect of STX on the ultrastructural features of *T. spiralis* muscle larvae compared to ABZ is examined by scanning electron microscopy (SEM)^28,29^. The isolated larvae were incubated with different treatments of STX and ABZ as described in the aforementioned in vitro experiment. After 24 h of inoculation, *T. spiralis* muscle larvae were harvested and transferred to a freshly prepared fixative solution containing 2.5% glutaraldehyde (w/v) in 0.1 M sodium cacodylate. The mixture was maintained at a pH of 7.2 at 37 °C and allowed for overnight incubation. Before being post-fixed in a solution of 2% (w/v) osmium tetroxide in sodium cacodylate buffer for 1 h, larvae were rinsed in 0.1 M sodium cacodylate buffer at pH 7.2 for 5 min. Liquid carbon dioxide was used to dry the specimens after dehydrating in an ethanol succession. The process of applying and affixing the desiccated larvae onto stubs equipped with a dual-sided carbon adhesion tape was executed prior to the gold coating using a sputtering coater (EIKO Engineering CO, Japan). The samples were then analyzed using a SEM (Jeol GSM-IT200, Japan) operating at 5 to 20 kV, Electronic Microscope Unit, Institute of Nanoscience and Nanotechnology, Kafrelsheikh University, Egypt.

## In vivo antiparasitic evaluation

### Drug administration

A 100 mg/kg/day dose of purified STX, ABZ, and STX niosomes was given. A 50 mg/kg/day dose of both STX niosomes and ABZ was given as a combination treatment. The required dose (250 µL) was administered orally for 3 consecutive days.

### Experimental design

Animals were divided into seven distinct groups (*n* = 10 per subgroup), as summarized in Table 2. Group I served as a negative normal control: uninfected and untreated. Group II was employed to evaluate the safety of STX. Group III served as a positive control, receiving infection without treatment. Group IV: infected and treated with purified STX pigment dissolved in 1% DMSO. Group V: infected and treated with ABZ. Group VI: infected and treated with STX niosomal formulation. Group VII: infected and treated with a combination therapy. Groups were further subdivided into two subgroups (I and M) to evaluate the impact of the administered drugs for three consecutive days during the intestinal phase (I) (3–5 days post-infection), and the muscular phase (M) (30–32 days post-infection).

**Table 2:** Experimental design and treatment protocol of different phases of *Trichinella spiralis* in mice.

Groups	Subgroups	Treatment protocol
Group I	------	Uninfected, receiving PBS with 1% DMSO
Group II	------	Uninfected, receiving STX
Group III	III_I_	Infected, untreated (control for intestinal phase)
	III_M_	Infected, untreated (control for muscle phase)
Group IV	IV_I_	Treated with STX, 2 days post infection
	IV_M_	Treated with STX, 30 days post infection
Group V	V_I_	Treated with ABZ, 2 days post infection
	V_M_	Treated with ABZ, 30 days post infection
Group VI	VI_I_	Treated with STX niosomes, 2 days post infection
	VI_M_	Treated with STX niosomes, 30 days post infection
Group VII	VII_I_	Treated with combination therapy, 2 days post infection
	VII_M_	Treated with combination therapy, 30 days post infection

### Assessment of parasite burden

On the specified termination day, all animals experienced an overnight fasting period and were subsequently sacrificed via intraperitoneal anesthesia using urethane (1.3–1.5 g/kg in approximately 1.5 g/5 mL of 0.9% normal saline solution; Sigma-Aldrich, St. Louis, MO). Subgroup I (intestinal phase) involved scarifying mice on day 6 post-infection, processing the small intestine according to previously instructions, obtaining adults of *T. spiralis*, and calculating the worm reduction rate. In addition, the mice in subgroup M (muscular phase) were sacrificed on 35 post infection, and the pepsin digestion technique was used to retrieve the muscle larvae. The McMaster counting chamber, Faust-Germany, was used for the microscopic counting of the larvae^4,24,30^.

### Histopathological examination

The studied groups’ intestinal and skeletal muscle segments were fixed in 10% formalin for 24 h, rinsed in water for 12 h, dehydrated in increasing alcohol grades, and then cleaned in xylene. For two h at 55 °C, impregnation was carried out in pure soft paraffin. A microtome was used to cut ten hard paraffin sections that were 5 μm thick. Haematoxylin and eosin stain was applied to the sections. The presence or absence of Trichinella larvae, any pathological abnormalities, and the degree of inflammatory cellular response were assessed histopathologically in intestinal sections. Skeletal muscle sections were inspected for Trichinella cysts, and comments regarding the capsule’s integrity, content, and inflammatory cellular response were recorded for each group^27^.

### Immunohistochemical examination

Skeletal muscle specimens’ paraffin sections were deparaffinized and rehydrated. Microwaving the slices in a citrate buffer (pH 6.0) allowed the antigen to be recovered. The endogenous peroxidase was inhibited using methanol that contained 3% hydrogen peroxide. Sections were incubated with the primary antibodies, Anti-CD34 Antibody (Supplier: DAKO, Catalog Number: M7165, Clone: QBEnd-10, Mouse anti-Human), in an ideal dilution of 1:50–1:100 for an overnight at 4 °C in a humid chamber. The secondary antibody, Biotin streptavidin link, DAKO, was then applied. To further pinpoint the antigen’s location, a chromogen solution of 3,3’-diaminobenzidine tetrahydro chloride (DAB) substrate was added (Universal Detection Kit, DAKO Envision, Denmark). Slides were then mounted, dehydrated in alcohol, and counterstained with hematoxylin^4^.

### Histopathological and immunohistochemical scoring

Small intestine sections were histologically scored using a semiquantitative scoring approach that assigned grades to the following characteristics: goblet cell hyperplasia (0, normal; 1, mild-only in crypts; 2, moderate-in crypts and villi); the presence of mast cells (0, absent or 1, present); eosinophils in epithelium and muscularis mucosae (0, absent or 1, present); plasma cells in lamina propria (0, absent or 1, present); crypt hyperplasia (0, absent or 1, present); villous atrophy (0, normal; 1, mild; 2 moderate and 3 severe)^31^. The inflammatory reaction encompassing the capsule was assessed and scored for the skeletal muscle specimens as follows: +1 denotes mild, + 2 moderate, and + 3 an extreme reaction^32^. The percentage of positive stained cells (quantity score) and the staining intensity score were combined to determine the immunohistochemistry score (IHS). Scores were assigned to the intensity of the staining: 0 (negative), 1 (mild), 2 (moderate), and 3 (strong). The quantity score was calculated on a scale of 0 to 4, as indicated below: 0 indicates that there is no immunostaining; 1 indicates that 1–10% of the cells are positive; 2 indicates that 11–50% of the cells are positive; 3 indicates that 51–80% of the cells are positive; and 4 indicates that 81–100% of the cells are positive. To calculate the IHS, the quantity score (0–4) was multiplied by the staining intensity score (0–3), which ranged from 0 to 12. Strong immunoreactivity was defined as a score of 9–12 (+ 3), moderate immunoreactivity as 5–8 (+ 2), feeble immunoreactivity as 1–4 (+ 1), and negative immunoreactivity as 0^32^.

### Computational inspection of STX binding affinity for the parasite’s essential proteins

The binding efficiency of STX for blocking essential proteins involved in different stages of *T. spiralis* life cycle, including *T. spiralis* β-tubulin, calreticulin (Ts-CRT), and cathepsin F (Ts-CF1), was examined in silico^4,29^. From the RCSB protein data repository, the model of β-tubulin protein’s X-ray crystallographic structure bound to albendazole sulphate (PDB ID: 1OJ0) was obtained (https://www.rcsb.org/). Due to the lack of crystallographic structures for *T. spiralis* calreticulin and cathepsin F, homology modeling was generated for the prediction of the 3D protein structures.

### Structure prediction and validation of target proteins

The NCBI GenBank database provided the *T. spiralis* calreticulin and cathepsin F amino acid sequences in the FASTA format. Their respective GenBank accession numbers are KAL1242782.1 and XP_003378245.1, respectively. The “Easy Modeller 4.0” tool in the “Threading Assembly Refinement (I-TASSER)” software was used to implement homology modeling iteratively. Templates and binding sites for these sequences were identified using the I-TASSER service (http://zhanglab.ccmb.med.umich.edu/I- TASSER/). Utilizing the LOMETS tool for template-based protein structure prediction within the I-TASSER, homology modeling of the mature domain of Ts-CF1 was conducted, employing the wild-type human procathepsin K crystal structure (PDB ID: 7PCK) as a template, with a Z-score of 4.39. Furthermore, the human calreticulin arm domains crystal structure (PDB ID: 3RG0), with a Z-score of 6.40, was used as a template for constructing the 3D *T. spiralis* calreticulin structure. The best model with a confidence score (C-score), which gauges the caliber of the anticipated model and prospective energy, was chosen for more research out of the five models of each protein that were generated by I-TASSER. The parameters produced during structure assembly simulations, the template that was employed, and the effects of threaded structure alignment were the basis for the computed C-score. The C-score typically ranges from − 5 to 2^33^. Structure analysis and verification server version 6 (SAVES v6.0) was used to authenticate the target proteins’ best validation structures. The 3D sequence profile for protein models was tested using the scores of the VERIFY-3D and ERRAT tools using SAVES v6.0 outputs, and the PROCHECK tool was utilized to confirm structure using the Ramachandran plot. PROCHECK analyzes the overall structural geometry and residue-by-residue geometry to determine the stereochemical nature of a protein structure^34^.

### Protein interaction and molecular Docking analysis

Molecular Operating Environment (MOE, 2020.09) was used to conduct the molecular docking investigations. The partial charges were automatically computed using the MMFF94x force field after all minimizations were carried out using MOE up to a Root Mean Square Deviation (RMSD) gradient of 0.05 kcal mol − 1 Å−1. Using the protonate 3D protocol in MOE with its default settings, the protein chains under investigation were prepared for molecular docking simulations by adding polar hydrogens and deleting all residues, creating protonation states that were advantageous for the simulations. Docking pose building was accomplished using the London-dG scoring function and the Triangle Matcher placement approach^4,29^.

### Statistical analysis

Statistical analysis was performed using GraphPad Prism 5. The results were presented as the mean ± standard deviation and statistical significance was set at *P*-value < 0.05. The significance of the differences between the experimental and control groups was analyzed by the student’s t-test. The in vitro experiments were analyzed using repeated measures ANOVA with post *hoc* Bonferroni test. One-way ANOVA was used to analyze the statistical significance between the in vivo groups with Tukey’s test as post *hoc* for pair wise comparison. Histopathological and immunohistochemical scoring were analyzed using Kruskal-Wallis test and Dunn’s post-test.

## Results

### Drug-likeness and ADMET predictions

**Fig. 1:**
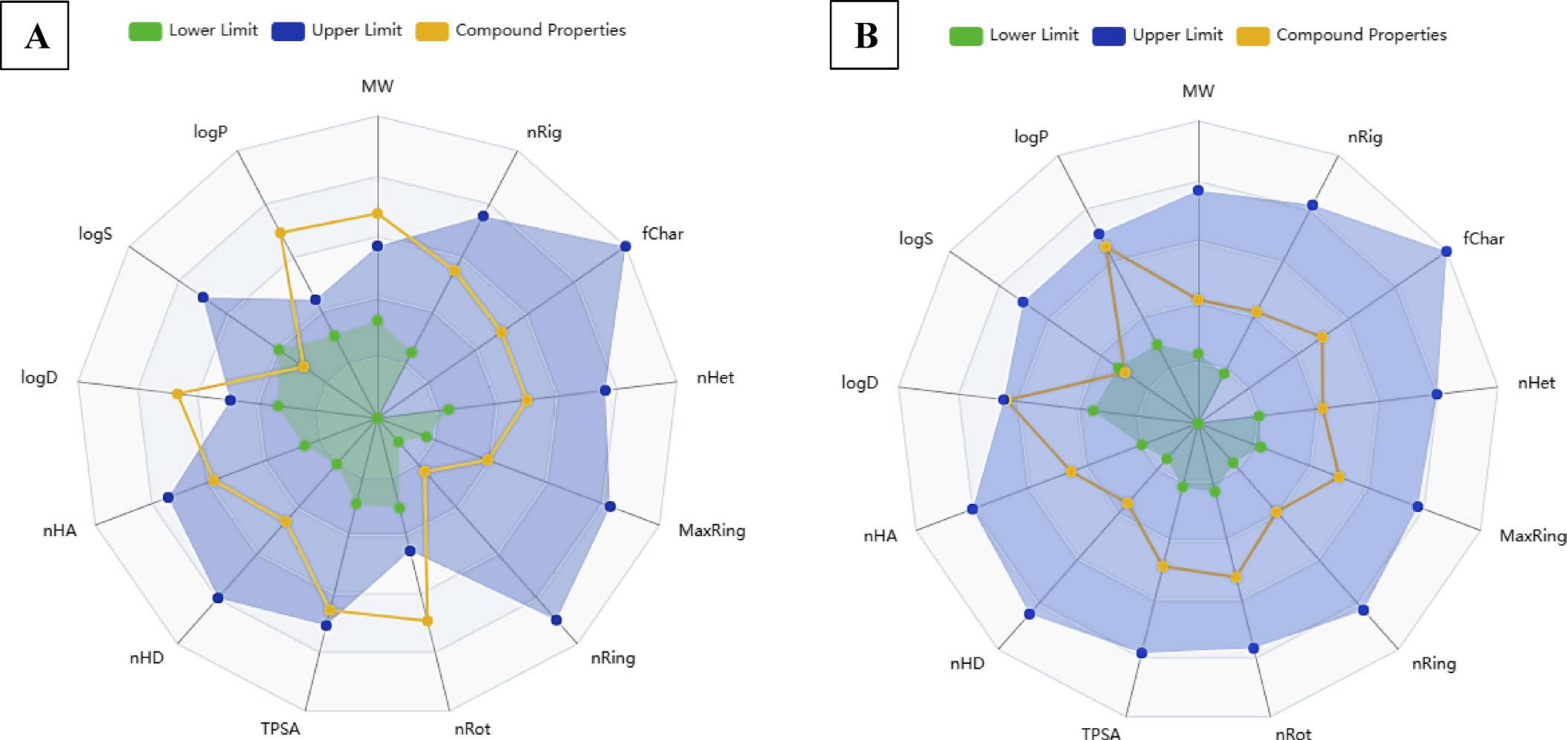
Radar charts of physicochemical properties predictions of STX (**A**) and ABZ (**B**). logP: logarithm of the n-octanol/water distribution coefficient at pH = 7.4; logS: logarithm of the aqueous solubility value; logD: logarithm of the n-octanol/water distribution coefficient; nHA: number of hydrogen bond acceptors; nHD: number of hydrogen bond donors; TPSA; topological polar surface area; nRot: number of rotatable bonds; nRing: number of rings; MaxRing: number of atoms in the biggest ring; nHet: number of heteroatoms; fChar: formal charge; nRig: number of rigid bonds; MW: molecular weight.

The drug-likeness of STX was assessed by ADMETlab 3.0 predictions of physicochemical properties, as shown in Fig. 1. Lipinski’s rule is utilized to determine the permeability and solubility using five parameters. Compounds that contravene Lipinski’s Rule of Five (RO5) are more prone to demonstrate inadequate absorption or penetration. The analysis revealed that STX exhibited two violations, whereas ABZ did not violate Lipinski’s rule. Number of hydrogen bond acceptors (nHA), hydrogen bond donors (nHD), and topological polar surface area (TPSA), of STX are predicted to be in the optimal range. However, the molecular weight and octanol/water partition coefficient (Log P_o/w_) of STX exceed the optimal values, suggesting poor absorption and oral drug-likeness administration.

It is vital for drug discovery efforts to target both pharmacological action and pharmacokinetics features. Human intestinal absorption (HIA), madin-darby canine kidney (MDCK) cells permeability, cancer coli-2 (Caco-2) cells permeability, and parallel artificial membrane permeability assay (PAMPA) were among the various criteria used to evaluate STX’s permeation capacity. The results displayed in Table 3 revealed that the Caco-2 permeability model of STX was found to be close to the acceptable range, while MDCK and PAMPA models showed low passive permeability (< 2 × 10^−6^ cm/s). STX showed HIA value of < 30% lower than HIA value of ABZ (> 30%). The distribution and partitioning in body tissues were also predicted. The plasma protein binding (PPB) affinity of STX is the optimal range (87.908%), even more than ABZ (85.36%). STX exhibited minimal BBB permeability (0.001), which elucidates its limited capacity to traverse the blood-brain barrier (BBB) and induce neurotoxicity. Moreover, the findings indicate that STX exhibits a negative predictive capacity concerning the inhibition of the CYP family (CYP1A2, CYP2C9, CYP2D6, and CYP3A4), as shown in Table 3. The anticipated excretion parameters, characterized by half-life (T½) and plasma clearance (CL), indicated that STX demonstrated a short half-life of 1.342 h and a low clearance rate of 2.33 ml/min/kg. The toxicological properties presented in Table 4 are crucial pharmaceutical considerations, encompassing mutagenicity as assessed by the ames test and carcinogenicity. The information gathered points to STX as non-mutagenic and suggests a negative prediction of genotoxicity. The acquired data indicate a negligible probability of detrimental impacts from the studied STX on cardiac rhythm, as evidenced by the minimal inhibition of the human ether-a-go-go related gene in comparison to ABZ. The ADMET predictions further suggest that STX exhibits a lower index of rat oral acute toxicity (ROA), neurotoxicity, hepatotoxicity, respiratory toxicity, and immunotoxicity, juxtaposed with ABZ.

**Table 3:** Prediction of Pharmacokinetic parameters of the tested drugs.

Absorption						
Comp.	Caco-2 Permeability	MDCK Permeability	PAMPA	Pgp-inhibitor	Pgp-substrate	HIA
STX	−5.129	−4.821	0.484	0.003	0.975	0.014
ABZ	−4.994	−4.631	0.027	0.524	0.05	0.137

Distribution					Excretion	
Comp.	PPB	VDss	BBB	BCRP inhibitor	CL_plasma_	T_1/2_
STX	87.908	−0.107	0.001	0.004	2.33	1.342
ABZ	85.36	−0.071	0.933	0.011	3.166	0.844

Metabolism						
Comp.	CYP1A2 inhibitor	CYP1A2 substrate	CYP2C19 inhibitor	CYP2C19 substrate	CYP2C9 inhibitor	CYP2C9 substrate
STX	0.018	0.0	1	0.0	0.185	0.033
ABZ	1	1	0.0	0.059	0.0	0.863

Comp.	CYP2D6 inhibitor	CYP2D6 substrate	CYP3A4 inhibitor	CYP3A4 substrate	HLM Stability	
STX	0.177	0.001	0.025	0.0	0.977	
ABZ	0.0	0.034	0.0	0.996	0.893	

PAMPA: parallel artificial membrane permeability assay; PPB: plasma protein binding; HIA: human intestinal absorption; BBB: blood brain barrier; VDss: volume distribution; BCRP: breast cancer resistance protein; HLM: human liver microsomal stability.

**Table 4:** Prediction of toxicity profile of the tested drugs.

Toxicity	STX	ABZ	Toxicity	STX	ABZ
hERG Blockers	0.107	0.283	Respiratory	0.161	0.964
hERG Blockers (10 μm)	0.647	0.629	Human Hepatotoxicity	0.487	0.806
DILI	0.413	1.0	Drug-induced Nephrotoxicity	0.92	0.986
AMES Mutagenicity	0.389	0.713	Ototoxicity	0.887	0.738
Rat Oral Acute Toxicity	0.097	0.36	Hematotoxicity	0.233	0.926
FDAMDD	0.107	0.68	Genotoxicity	0.0	0.999
Skin Sensitization	0.999	0.63	RPMI-8226 Immunitoxicity	0.204	0.402
Carcinogeni city	0.208	0.924	A549 Cytotoxicity	0.526	0.947
Eye Corrosion	0.0	0.0	Hek293 Cytotoxicity	0.046	0.972
Eye Irritation	0.314	0.511	Drug-induced Neurotoxicity	0.013	0.282

hERG: human ether-a-go-go related gene; FDAMDD: FDA Maximum (Recommended) Daily Dose; DILI: drug induced liver injury. Values:0–0.3 (high),0.3–0.7(medium), and0.7–1.0(poor).

### Characterization of the formulated niosomes

**Fig. 2:**
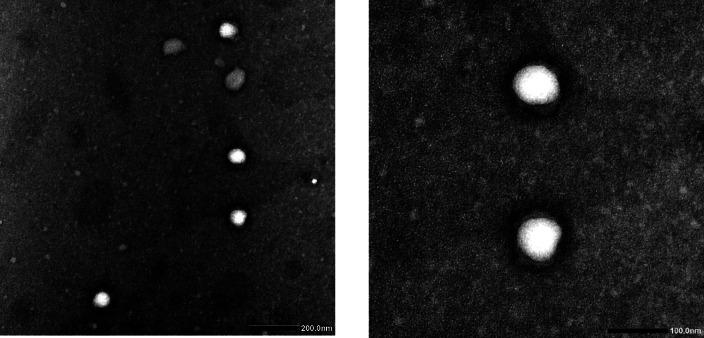
Representative TEM micrographs of niosomes showing spherical vesicles within the nanoscale range.

The niosomal formulation’s morphology, zeta potential, and particle size were assessed. Representative TEM micrographs of the formulated niosomes are displayed in Fig. 2. The micrographs depict spherical particles exhibiting diameters within the nanoscale range. DLS measurement provided further verification of the vesicle size, showing that it was in the nanoscale range with an average size of 177.8 nm. Noteworthy, according to the recorded polydispersity index value (0.383), the produced niosomes exhibited a consistent vesicle size. Our findings revealed that the zeta potential of the prepared niosomes is a high negative value of −35.23 ± 1.26, indicating the strength and stability among the niosomal vesicles. The encapsulation efficiency of STX into niosomal vesicles is 92.7%. The estimation of drug loading involved calculating the amount of drug encapsulated per gram of lipid. The results revealed that 185.4 mg of STX was loaded per gram of lipid in the formulation. Moreover, the stability of niosomes was assessed over a 30-day period under storage conditions of ambient temperature and refrigeration (4 °C). The results of our study indicated that niosomes stored in a refrigerator at 4 °C exhibited superior stability, as no discernible sedimentation or creaming was observed during the storage period. Additionally, the niosomal suspension maintained its initial particle size and EE%. Conversely, the niosomes that were stored at a temperature of 25 °C exhibited comparable changes in their initial particle size (836.2 nm; supplementary material Fig. S1) and EE% (85.3%).

### In vitro anthelmintic activity

**Fig. 3:**
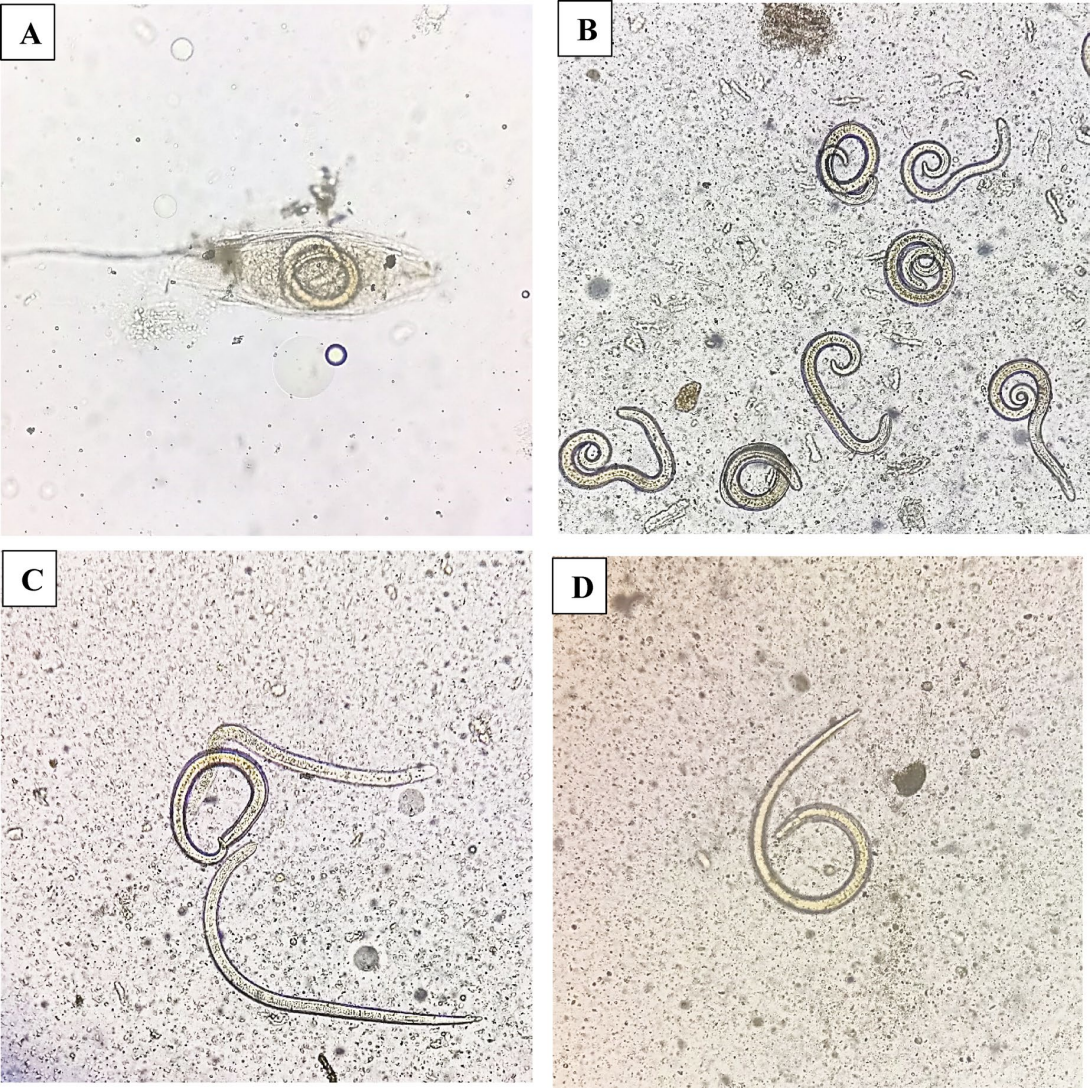
Representative photomicrographs of viability and mortality of *T. spiralis* muscle larvae. (**A**) encapsulated ML before treatment. (**B**) viable ML showing coiling movements. (**C**) straight immotile ML. (**D**) comma-shaped immotile ML.

The anthelmintic activity against *T. spiralis* muscle larvae using different treatments of purified STX pigment, STX niosomal formulation, and combination therapy was studied in vitro compared to the reference drug ABZ. Survival numbers and mortality rates of *T. spiralis* muscle larvae after treatment are demonstrated in Table 5. The assessment of viability was conducted by observing the movement and coiling behaviors of muscle larvae, as the presence of immotile, straight, or comma-shaped larvae is a characteristic indicator of mortality, as shown in Fig. 3. The results displayed in Table 5 indicated that the effect of different treatments on muscle larvae survival is contingent upon exposure time. The statistically significant (*P* < 0.05) impact of all treatments was apparent from the first hour of incubation. Complete eradication of muscle larvae was observed after 48 h of incubation for niosomes and combinational therapy, while STX showed a mortality rate of 93.75%. Incubation of muscle larvae with STX pigment for 1 h showed higher killing activity and mortality rate (10.8%) compared to delayed-in-vitro-release niosomes (8%). Nevertheless, after 6 h of inoculation, niosomes demonstrated higher activity and mortality rate (30.83%) compared to mortality rates of STX (16.66%) and ABZ (22.5%). After 24 h of inoculation, all the tested agents showed significant activity (*P* < 0.05) and remarkable larvicidal control. Regarding to combination therapy, the synergistic effect between niosomes and ABZ was observed, as attested by higher mortality rates at all the duration of exposure compared to other groups.

**Table 5:** Survival numbers and mortality rates of muscle larvae exposed to different treatments at different time intervals.

Drugs	Exposure time (h)			
	1 h	6 h	24 h	48 h
Control	59.750 ± 0.500 (0.41%)	58.500 ± 1.290994 (2.5%)	53.250 ± 1.707825 (11.25%)	49.750 ± 1.707825 (17.08%)
STX	53.500 ± 1.29099^a^ (10.8%)	50.000 ± 2.16024^b^ (16.66%)	23.750 ± 3.77491^c^ (60.41%)	3.750 ± 2.98607^d^ (93.75%)
ABZ	51.250 ± 1.500^a^ (14.5%)	46.500 ± 2.38047^b^ (22.5%)	12.750 ± 1.70782^c^ (78.75%)	0.000 ± 0.000^d^ (100%)
Niosomes	55.250 ± 1.70782^a^ (8%)	41.500 ± 2.64575^b^ (30.83%)	9.750 ± 1.89296^c^ (83.75%)	0.000 ± 0.000^d^ (100%)
Combination	49.000 ± 1.41421^a^ (18.33%)	37.750 ± 2.21735^b^ (37.08%)	2.750 ± 1.70782^c^ (95.41%)	0.000 ± 0.000^d^ (100%)

The survival numbers of ML are presented as mean ± SD. Mortality rates are expressed in parenthesis. The statistical significance at *P* < 0.05 are represented by letters (a, b, c, and d) compared to control at each time interval (repeated measures ANOVA; *n* = 4).

### Ultrastructural alterations of the cultured *T. spiralis* larval stage

**Fig. 4:**
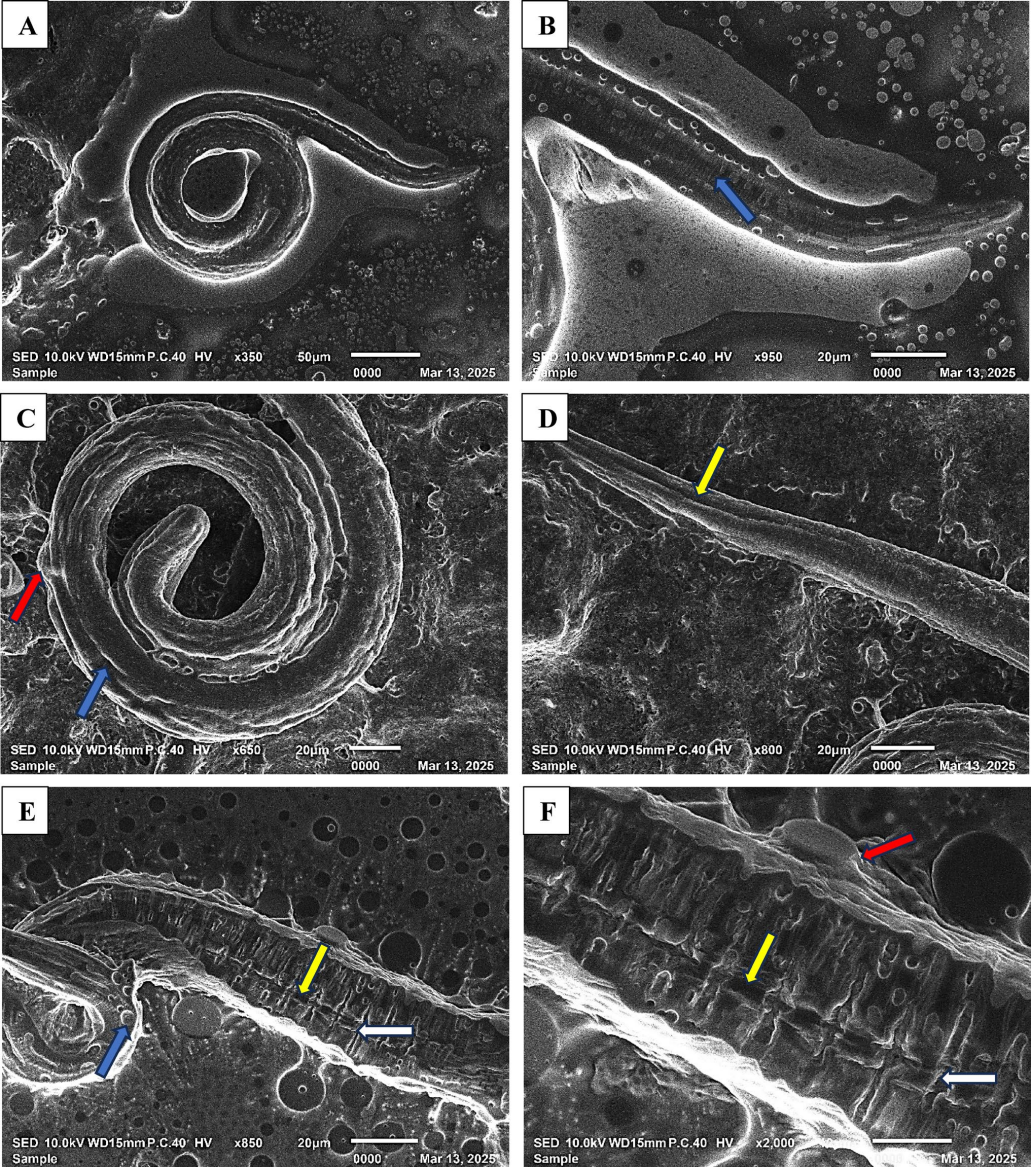
SEM micrographs demonstrating the effect of STX treatment on cultured *T. spiralis* muscle larva compared to ABZ. (**A**, **B**) Normal grown *T. spiralis* larva without treatment showing normal trunk with the typical structure of cuticle annulation, characterized by fine transverse fissures (blue arrow) and an array of bacillary apertures. (**C**, **D**) ABZ-treated *T. spiralis* larva showing destructed shrunken cuticle characterized by notches (blue arrow), blebs (red arrow), loss of its normal annulations, and shallow longitudinal fissure (yellow arrow). (**E**, **F**) STX-treated *T. spiralis* larva showing degenerative deformities of cuticle with the appearance of notches (blue arrow), membrane blebs with a large cauliflower mass (red arrow), and massive transverse (white arrow) and longitudinal fissures (yellow arrow).

SEM findings revealed that the muscle larvae incubated in RPMI medium without treatment showed an array of bacillary apertures, normal cuticle appearance, and a typical coiled structure, featuring fine transverse patterns and clearly defined annulation, as shown in Fig. 4. Concerning the impact of STX on cultured larvae, it was observed that there were excessive cuticular deformities, characterized by the appearance of a large mass with a cauliflower shape, numerous notches, blebs, and massive transverse and longitudinal fissures. ABZ demonstrated a comparatively less impact on the cuticle when juxtaposed with STX. The results indicated a damaged cuticle, characterized by notches, blebs, shallow longitudinal fissures, and a loss of its customary annulations.

### In vivo evaluation of anthelmintic activity

The anti-parasitic activity of purified STX pigment, STX niosomal formulation, and combination therapy was further evaluated in an in vivo study. The experiment was compared to the reference drug ABZ, in order to validate the prospective in vitro findings. During the intestinal phase, all treatment groups exhibited a significantly lower mean number of adult worms in the small intestine (*P* < 0.001) compared to the infected untreated group (85.33 ± 6.02). The results presented in Table 6 demonstrated that the efficacy of STX treatment on the infected mice (G IV_I_) was 65.62%, resulting in a significant reduction (*P* = 0.0002) in the mean adult worm count (29.33 ± 4.04) in comparison to the untreated infected group (G III_I_). Administration of ABZ suspension (G V_I_) significantly decreased the intestinal adult worm count (4.00 ± 2.00) with eliminating efficacy of 95.53% compared to groups G III_I_ and G IV_I_. Enhanced drug delivery of STX niosomal nanocarrier (G VI_I_) augmented the killing efficacy of STX up to 98.05%, and reduced the mean adult worms count (1.66 ± 1.52). Notably, the combination therapy administered to G VII_I_ successfully eradicated (100%) *T. spiralis* adult worms in infected mice, with no observable adult worms (0.00 ± 0.00).

In the muscular phase, the average count of muscle larvae per mouse was recorded as a measure of treatment efficacy. The results displayed in Table 7 revealed that all treated groups demonstrated a significant decrease in mean larvae count (*P* < 0.001) compared to the infected untreated group (32900 ± 1708.801). The combination therapy administered to group G VII_M_ showed the highest efficacy (90.73%) of eliminating the muscle larvae and a significant reduction of mean count (3047.66 ± 145.55), followed by the niosomes-treated group (G VI_M_) (83.08%) and ABZ-treated group (G V_M_) (74.96%), with mean counts of 5563.66 ± 545.03 and 8235.66 ± 670.04, respectively. In contrast, the STX-treated group (G IV_M_) showed the lowest efficacy (40.02%) and capacity of drug delivery, with a mean count of muscle larvae of 19733.33 ± 1159.02 compared to other treated groups.

**Table 6:** Survival numbers and reduction percentage of *T. spiralis* adult worms in small intestine after treatment.

Intestinal phase		G III_I_ Positive control	G IV_I_ STX-treated	G V_I_ ABZ-treated	G VI_I_ Niosomes-treated	G VII_I_ Combination-treated
Mean ± SD (*n* = 5)		85.33334 ± 6.027714	29.33333 ± 4.041452	4.000 ± 2.000	1.666667 ± 1.527525	0.000 ± 0.000
Student’s ttest *P-*value		-	0.0002	< 0.0001	< 0.0001	< 0.0001
ANOVA		*P-*value = < 0.0001				
Post hoc test (Tukey’s) *P-*value	G III_I_	-	< 0.05	< 0.0001	< 0.0001	< 0.0001
	G IV_I_	-	-	< 0.0001	< 0.0001	< 0.0001
	G V_I_	-	-	-	Ns	Ns
	G VI_I_	-	-	-	-	Ns
	G VII_I_	-	-	-	-	-
% of reduction*		-	65.62%	95.53%	98.05%	100%

* % of reduction = [(survival mean count of group G III_I_ – survival mean count of the treated group)/survival mean count of group G III_I_] × 100.

Ns represents statistical non-significance.

**Table 7:** Survival numbers and reduction percentage of *T. spiralis* muscle larvae in skeletal muscles after treatment.

Muscular phase		G III_M_ Positive control	G IV_M_ STX-treated	G V_M_ ABZ-treated	G VI_M_ Niosomes-treated	G VII_M_ Combination-treated
Mean ± SD (*n* = 5)		32900.000 ± 1708.801	19733.330 ± 1159.023	8235.667 ± 670.045	5563.667 ± 545.0306	3047.667 ± 145.5553
Student’s ttest *P-*value		-	0.0004	< 0.0001	< 0.0001	< 0.0001
ANOVA		*P-*value = < 0.0001				
Post hoc test (Tukey’s) *P-*value	G III_M_	-	< 0.05	< 0.0001	< 0.0001	< 0.0001
	G IV_M_	-	-	< 0.0001	< 0.0001	< 0.0001
	G V_M_	-	-	-	< 0.0001	< 0.0001
	G VI_M_	-	-	-	-	Ns
	G VII_M_	-	-	-	-	-
% of efficacy		-	40.02%	74.96%	83.08%	90.73%

* % of reduction = [(survival mean count of group G III_M_ – survival mean count of the treated group)/survival mean count of group G III_M_] × 100.

Ns represents statistical non-significance.

### Histopathological examination

**Fig. 5:**
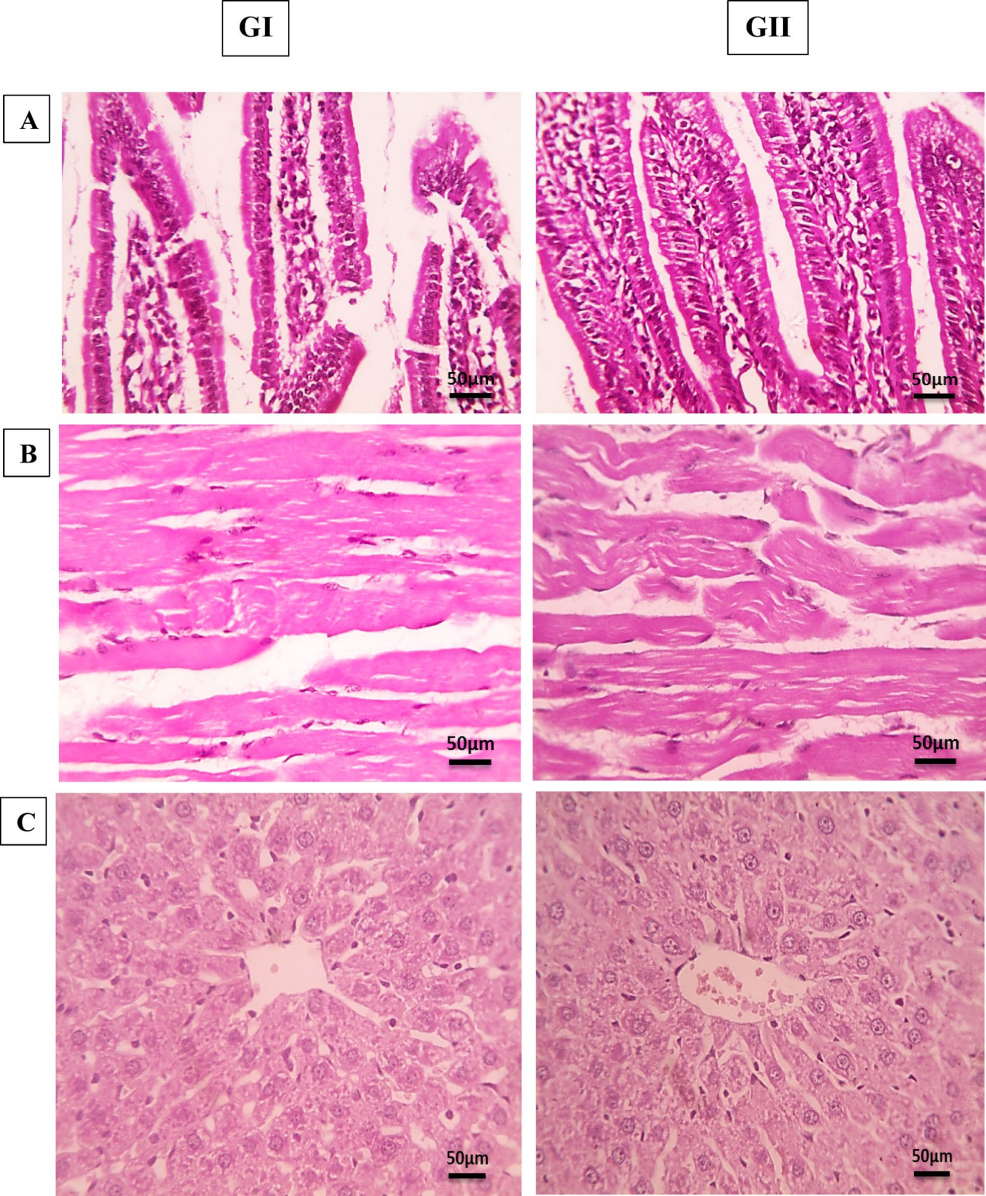

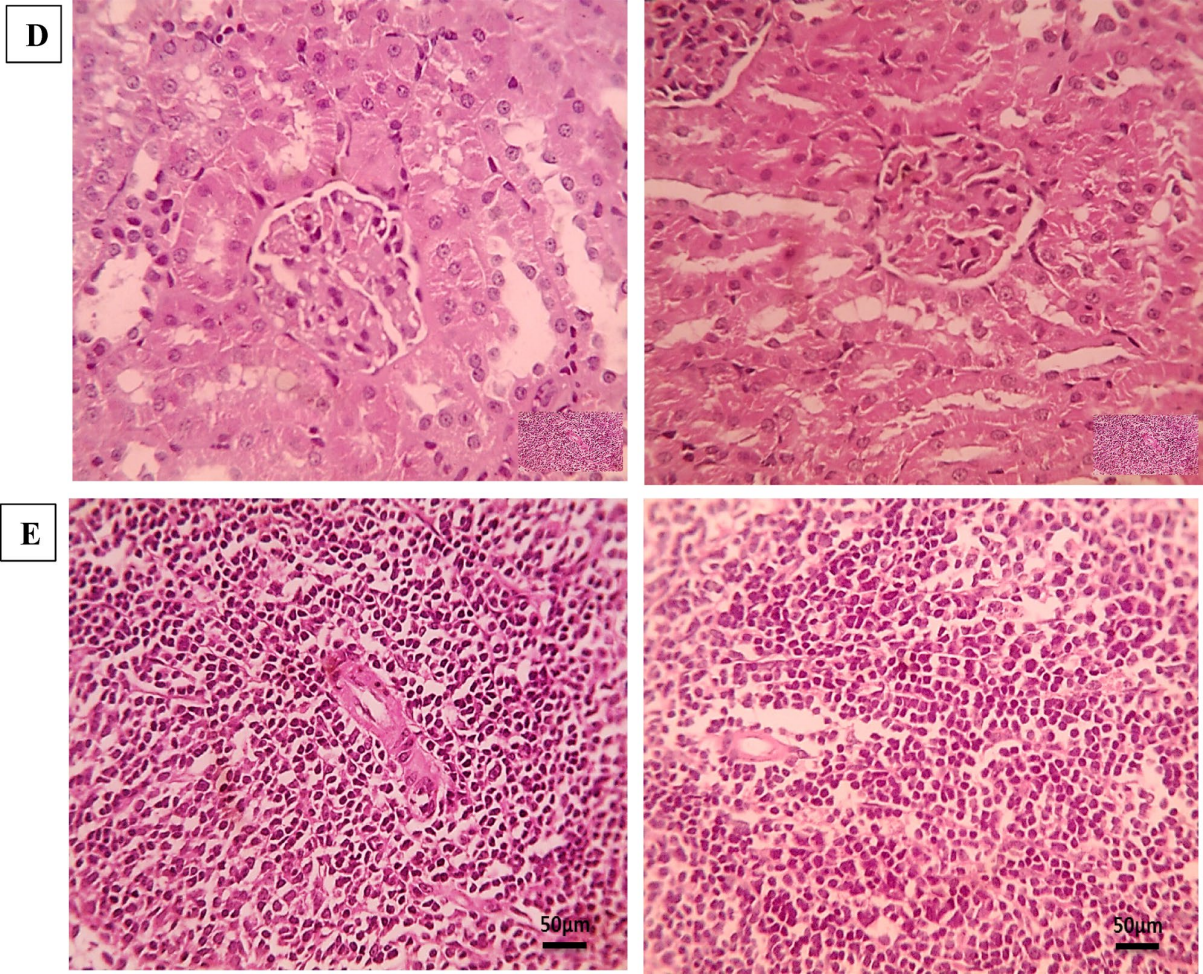
Effect of administered STX on body organs of group GII compared to normal control GI. (**A**) Photomicrographs of intestinal sections showing normal structure of villi, crypts, lamina propria, and muscles (400x). (**B**) Photomicrographs skeletal muscles sections showing normal structure of muscle fibers with no signs of inflammation or fibrosis (400x). (**C**) Photomicrographs of the liver section showing normal hepatic architecture where hepatic cords radially arranged around central veins with normal sinusoids (400x). (**D**) Photomicrographs of the kidney section showing normal glomeruli, tubules, and interstitial tissue (400x). (**E**) Photomicrographs of the spleen section showing normal splenic architecture with well-defined lymphoid follicles and normal red pulp (400x).

First, the toxicity of the tested STX pigment was evaluated in mice to validate the safety profile obtained by ADMET predictions and cytotoxicity on normal Vero cells. The results displayed in Fig. 5. revealed that STX administration to GII showed normal tissue findings with no pathological abnormalities compared to normal control (GI).

**Fig. 6:**
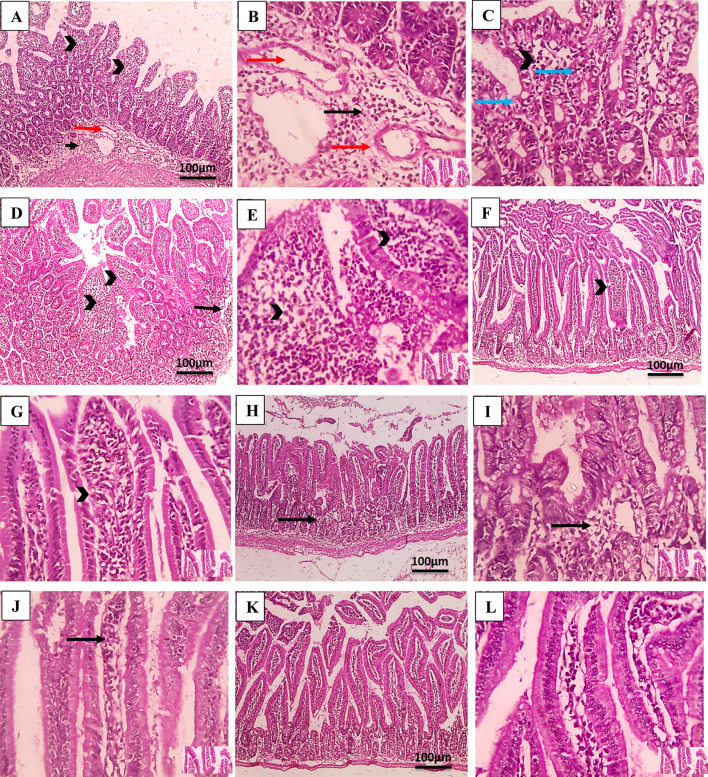

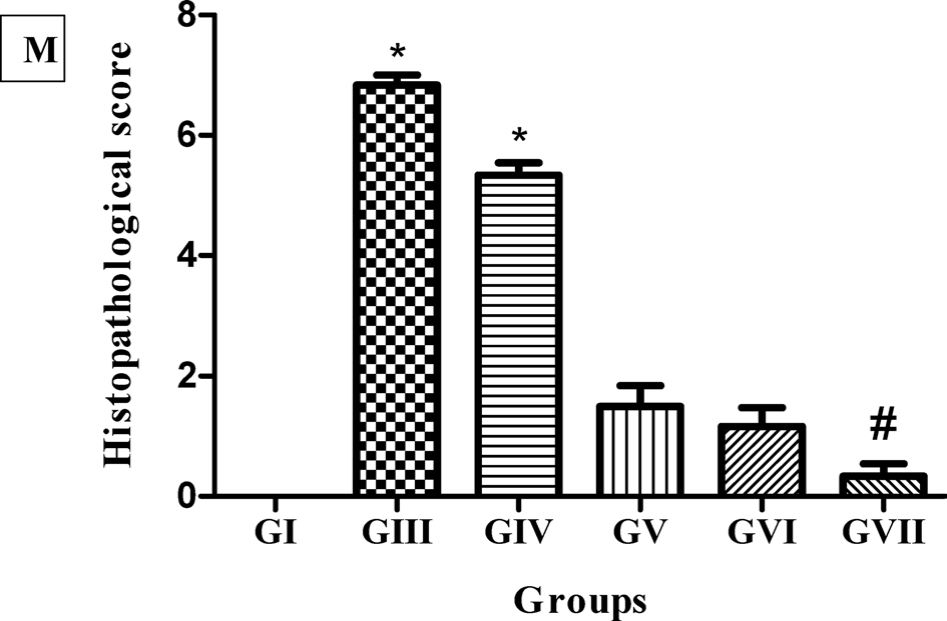
Histopathological findings of H&E-stained intestinal sections. (**A**; 100x), (**B**; 400x), and (**C**; 400x) infected untreated group (GIII) showing dense leukocytic cells infiltration (arrowhead), goblet cells hyperplasia (blue arrow) besides hyperplasia of the crypts (thick black arrow). (**D**; 100x) and (**E**; 400x) STX-treated group (GIV) showing shortened villi (arrowhead), crypts hyperplasia (thick black arrow), leukocytic cells infiltration (arrowhead). (**F**; 100x) and (**G**; 400x) ABZ-treated group (GV) showing fewer leukocytic cells infiltration (arrowhead). (**H**; 100x), (**I**; 400x), and (**J**; 400x) niosomes-treated group (GVI) showing few leukocytic cells infiltration (arrowhead). (**K**; 100x) and (**L**; 400x) combination treated group (GVII) showing normal crypts and normal lamina propria. (**M**) Histopathological lesion scores in small intestine (*n* = 6). * Means significant *p* < 0.05 when compared to normal control group G1. # means significant *p* < 0.05 when compared to GIII & GIV by Kruskal-Wallis test and Dunn’s post-test.

Photomicrographs of H&E-stained small intestine were examined to evaluate the efficacy of STX, niosomes, and combination therapy on *T. spiralis* infection during the intestinal phase compared to ABZ, as shown in Fig. 6. After 6 days of muscle larvae inoculation, histopathological findings of the infected untreated group (GIII) showed a dense infiltration of inflammatory cells, primarily in the villi’s core and extending into the submucosa with shortened villi and increased villous width. The villous inflammation is observed with dense leukocytic cell infiltration consisting mainly of eosinophils, neutrophils, plasma cells, and lymphocytes extending into the lamina propria. A further decline in the villous height to crypt depth ratio, along with obvious goblet cell hyperplasia in epithelial lining villi and crypts, further demonstrated the flattening of the villi and hyperplasia of the Lieberkühn crypts. The intestinal wall of the STX-treated group (GIV) showed a slight reduction of leukocytic cell infiltration in villi and lamina propria with shortened villi, increased villous width, and crypt hyperplasia. The ABZ-treated group (GV) sections exhibited a notable reduction in the intensity of the leukocytic cellular infiltration in the villous core with normal crypts and normal lamina propria. Photomicrographs of the niosomes-treated group (GVI) showed fewer leukocytic cell infiltrations in the lamina propria with elongated villi and goblet cell hyperplasia in epithelial lining crypts. Regarding to the examined sections of the combination-treated group (GVII), the intestinal wall demonstrated a significant decline of inflammatory cellular infiltration, alongside a restoration of the normal villous architecture, crypts, and lamina propria. Moreover, the semi-quantitative histopathological scoring revealed that the treated groups demonstrated improved scores compared to GIII, as presented in Fig. 6M.

**Fig. 7:**
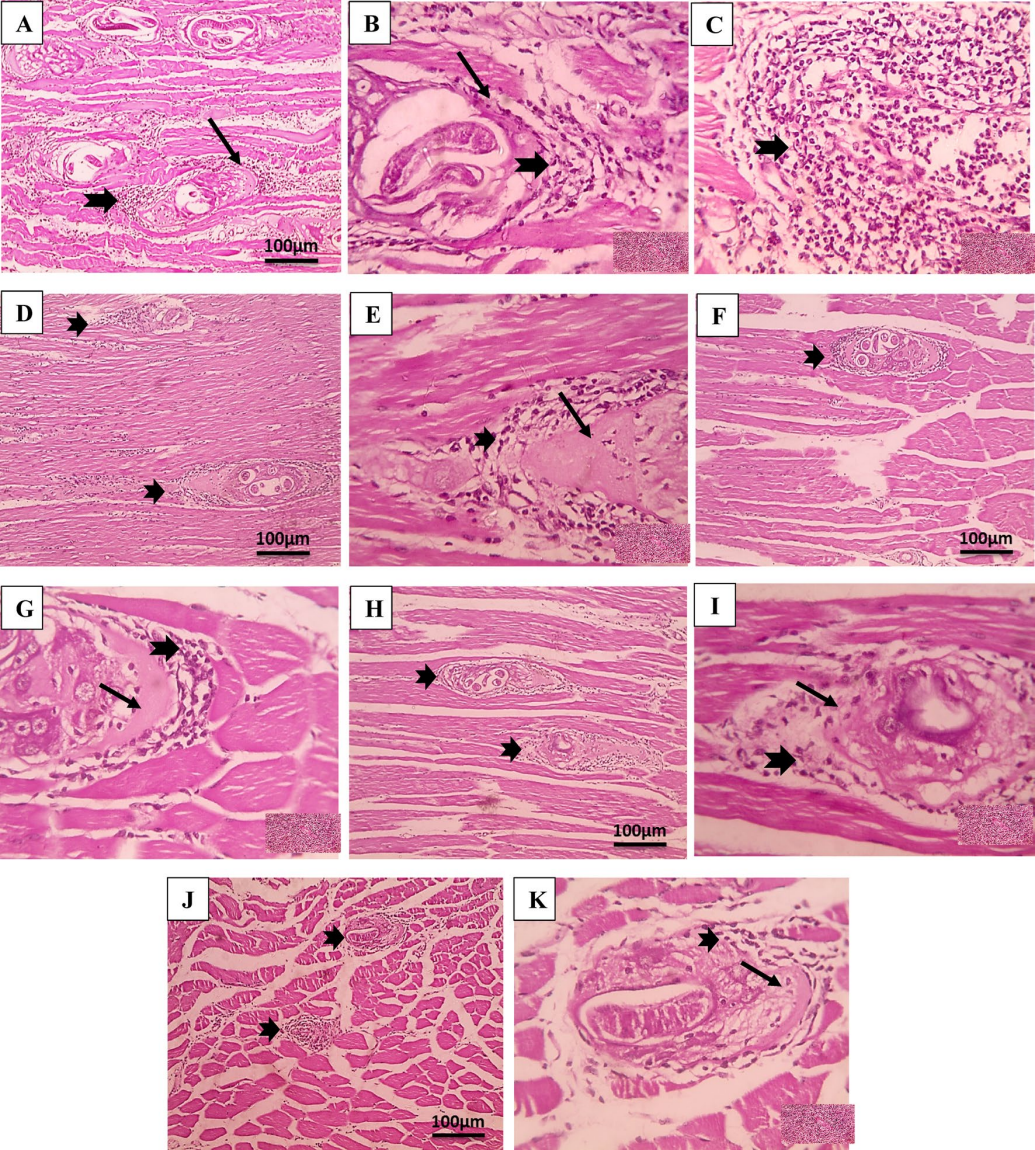

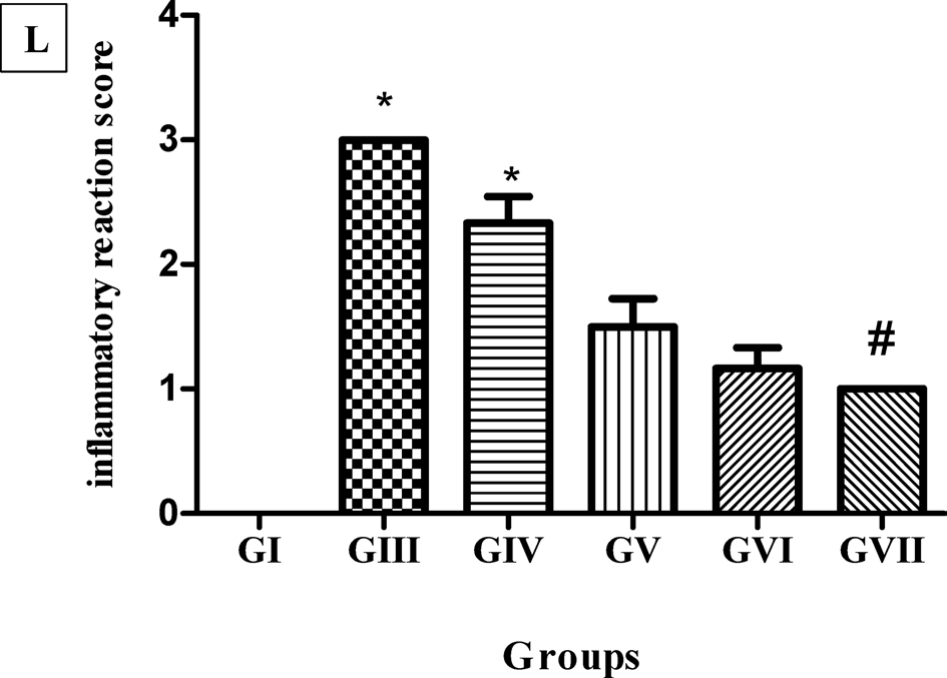
Histopathological findings of H&E-stained skeletal muscle sections. (**A**; 100x), (**B**; 400x), and (**C**; 400x) infected untreated group (GIII) showing many encysted trichnoid larvae (thin black arrow) and intense inflammatory infiltration (thick black arrow). (**D**; 100x) and (**E**; 400x) STX-treated group (GIV) showing numerous larval deposits (thin black arrow) and dense inflammatory infiltration. (**F**; 100x) and (**G**; 400x) ABZ-treated group (GV) showing reduced larval deposits (thin black arrow) and moderate inflammatory infiltration. (**H**; 100x) and (**I**; 400x) niosomes-treated group (GVI) showing few larval deposits (thin black arrow) and mild inflammatory infiltration. (**J**; 100x) and (**K**; 400x) combination-treated group (GVII) showing least number larval deposits (thin black arrow) and mild inflammatory infiltration. (**L**) Histopathological lesion scores of inflammatory reactions (*n* = 6). * Means significant *p* < 0.05 when compared to normal control group G1. # means significant *p* < 0.05 when compared to GIII & GIV by Kruskal-Wallis test and Dunn’s post-test.

Skeletal muscle sections were also examined to evaluate the efficacy of the tested drugs on the encysted larvae during the muscular phase, as shown in Fig. 7. In the infected untreated group, histopathological examination of muscle sections revealed various chronic inflammatory cells, including eosinophils, plasma cells, lymphocytes, and histiocytes penetrating muscle bundles and encompassing the encysted larvae. Additionally, there was a notable dissipate degeneration of the infected muscle and a numerous number of encysted *T. spiralis* larvae, each encased with the collagen capsule, dispersed throughout the muscles’ sarcoplasm. Following STX treatment, the infected animals’ muscles displayed entire larvae enclosed in clearly defined, intact capsules as well as pericapsular histio-lymphocytic inflammatory cells infiltrate. Muscle sections of ABZ-treated group revealed a notable presence of pericapsular inflammatory cellular infiltration, alongside a reduced number of trichina capsules exhibiting focal deterioration. STX delivery via noisome nanovesicles resulted in a small number of larval deposits within the modified nursing muscle fibers, accompanied by a less pronounced inflammatory infiltration. The most significant enhancement, characterized by a reduced presence of larval cysts, localized degeneration of capsules, and a milder inflammatory infiltrate, was observed in the context of combination therapy.

### Immunohistochemical examination

**Fig. 8:**
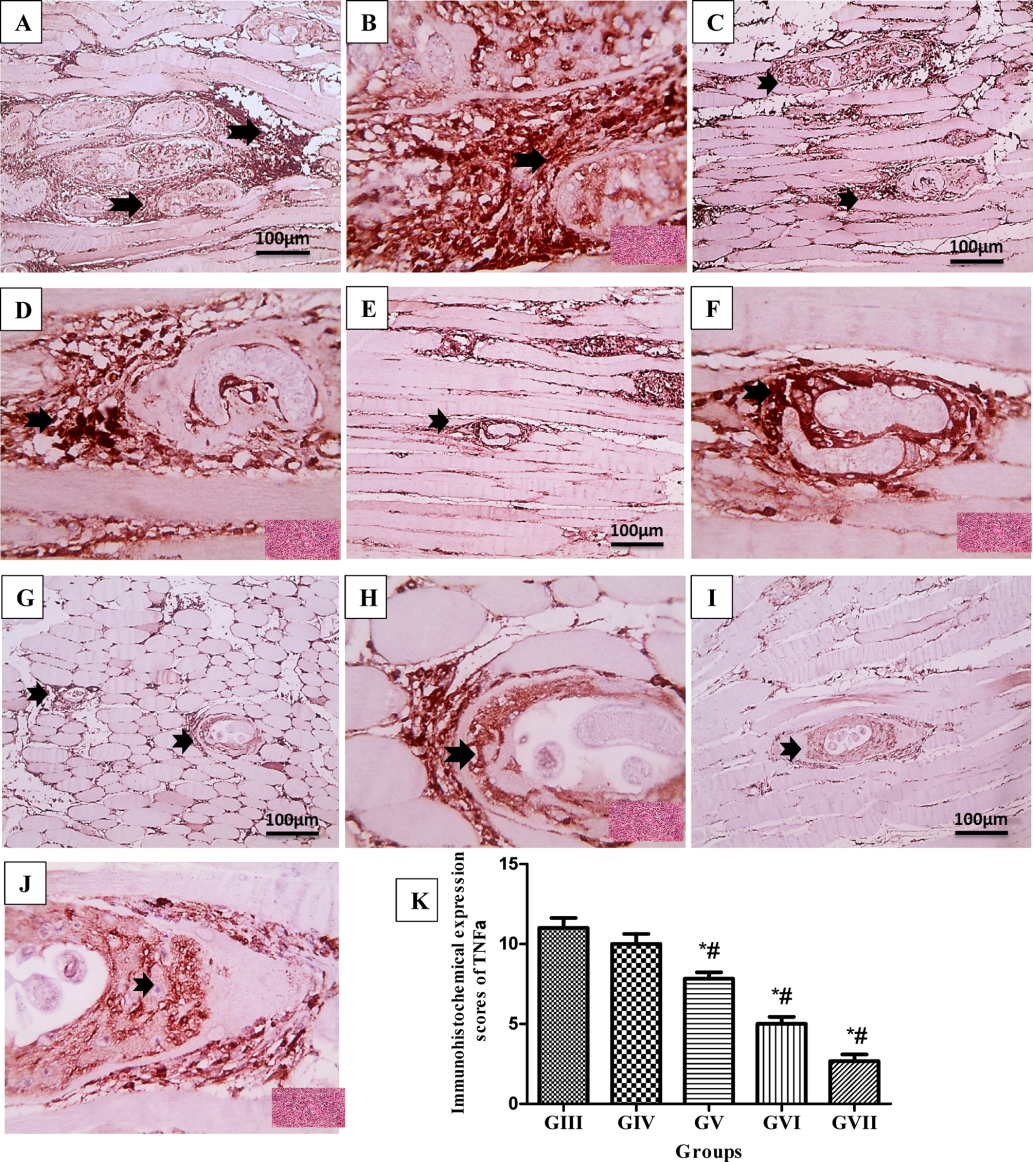
Immunohistochemical findings of TNFα-stained skeletal muscle sections. (**A**; 100x) and (**B**; 400x) infected untreated group GII showing strong cytoplasmic reaction against TNF-α (thick black arrow). (**C**; 100x) and (**D**; 400x) STX-treated group GIV showing strong cytoplasmic reaction against TNF-α (thick black arrow). (**E**; 100x) and (**F**; 400x) ABZ-treated group GV showing moderate cytoplasmic reaction against TNF-α (thick black arrow). (**G**; 100x) and (**H**; 400x) niosomes-treated group GIV showing moderate cytoplasmic reaction against TNF-α (thick black arrow). (**I**; 100x) and (**J**; 400x) combination-treated group GIV showing mild cytoplasmic reaction against TNF-α (thick black arrow). (**K**) Immunohistochemical scores of inflammatory reactions (*n* = 6). * Means significant *p* < 0.05 when compared to infected control group GIII. # means significant *p* < 0.05 when compared to GIV by Kruskal-Wallis test and Dunn’s post-test.

The immunostaining of the skeletal muscles to detect the expression of proinflammatory cytokine (TNF-α) was also employed to assess the efficacy of the tested treatments on *T. spiralis* infection during the encysted larvae stage. As illustrated in Fig. 8, T. *spiralis* infection revealed trichina capsules characterized by localized degeneration, encompassed by inflammatory cellular infiltration demonstrating strong expression of TNF-α, with a score of 11.00 ± 1.549 in the untreated control group. Skeletal muscle sections of the STX-treated group revealed trichina capsules encircled with marked infiltration of inflammatory cells demonstrating strong expression of TNF-α, with a score of 10.00 ± 1. 459. Treatment with ABZ greatly decreased the number of inflammatory cells in the trichina capsule of skeletal muscle compared to the infected control group, with a score of 7.833 ± 0.9832 showing moderate TNF-α expression. Loading STX in a niosomal nanocarrier significantly decreased the number of inflammatory cells infiltrating the trichina capsule of skeletal muscle compared to treatment with STX pigment, which showed moderate levels of TNF-α (with a score of 5.000 ± 1.095). The synergistic activity of the combination treatment was verified by immunostaining of skeletal muscle showing the highest alleviation of inflammatory cellular infiltration and mild expression of TNF-α, with a score of 2.667 ± 1.033.

### Homology modeling and validation of modeled proteins

**Fig. 9:**
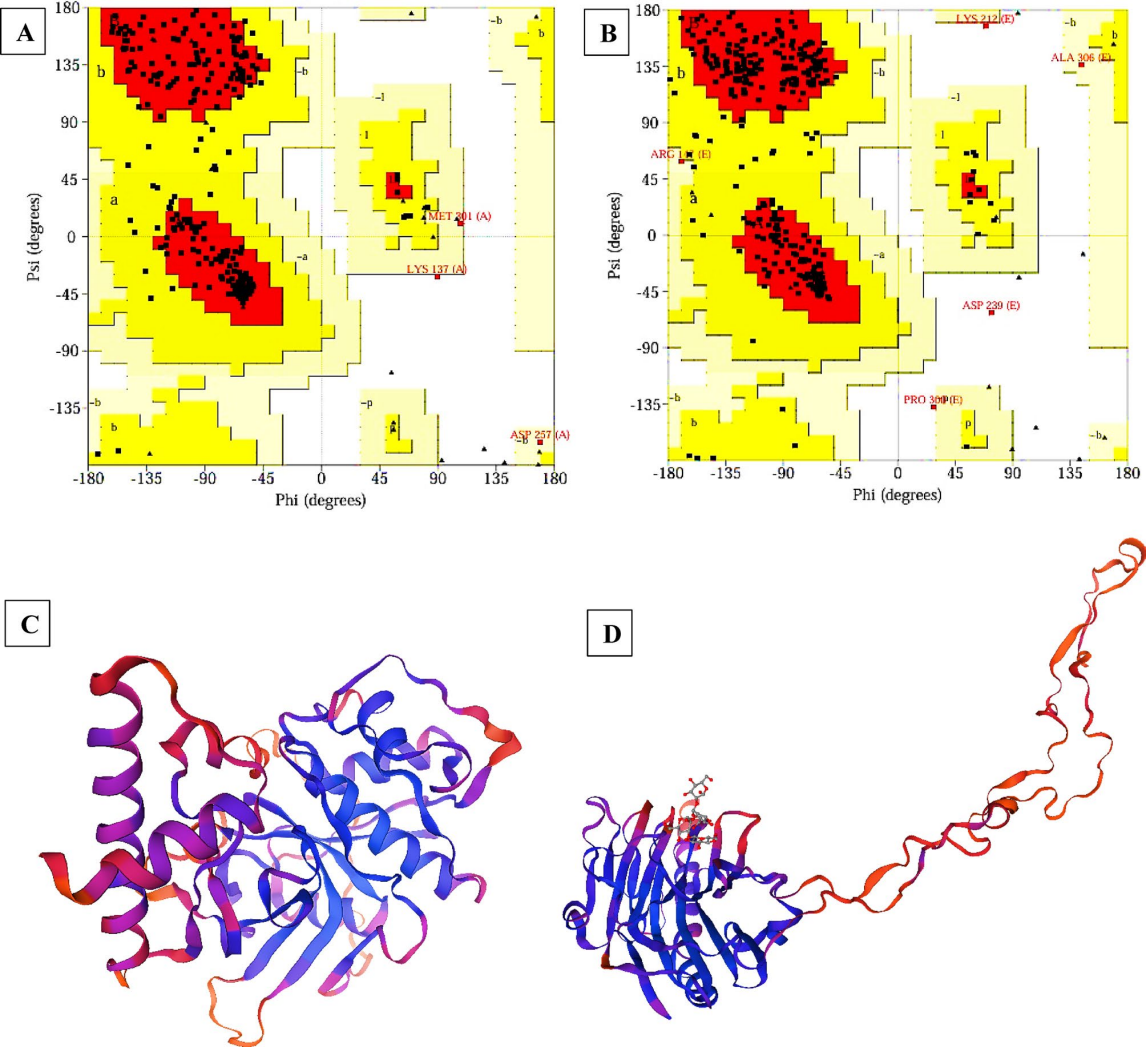
Homology modeling validation of *T. spiralis* target proteins demonstrating Ramachandran plots of Ts-CF1(**A**) and Ts-CRT (**B**), and 3D-modelled protein structures of Ts-CF1(**C**) and Ts-CRT (**D**).

The cluster density of each protein was used to construct five models using I-TASSER. The generated models exhibited varying confidence scores for each protein, and the models with the highest C-scores were selected for further analysis. For *T. spiralis* cathepsin F protein, the selected model, with the highest C-score of −1.52, displayed an estimated RMSD of 10.1 ± 4.6 Å and an estimated TM-score of 0.53 ± 0.15. The selected model for the *T. spiralis* calreticulin protein showed a C-score of 0.15, an estimated RMSD of 6.5 ± 3.9 Å, and an estimated TM-score of 0.73 ± 0.11. The selected models displayed the 3D-modeled protein structures, as shown in Fig. 9. Final validation of the refined structure: The SAVES v6.0 server was used to validate the refined I-TASSER structure. The Ramachandran plot statistics of *T. spiralis* cathepsin F model indicated that 87.8% of its residues are located in the most favored regions, 11.1% in additional allowed regions, 0.7% in generously allowed regions, and 0.4% in disallowed regions. The 3D-modelled *T. spiralis* calreticulin protein exhibited 85.2% of residues positioned within the most favored regions of the Ramachandran plot, alongside 13.5% in the additional allowed regions, 0.6% in the generously allowed regions, and 0.6% in the disallowed regions, as shown in Fig. 9. Lastly, Ts-CF1 and Ts-CRT were both modeled to a high standard, as evidenced by their overall quality factors of 94.412 and 98.196, respectively.

### Molecular Docking analysis

**Fig. 10:**
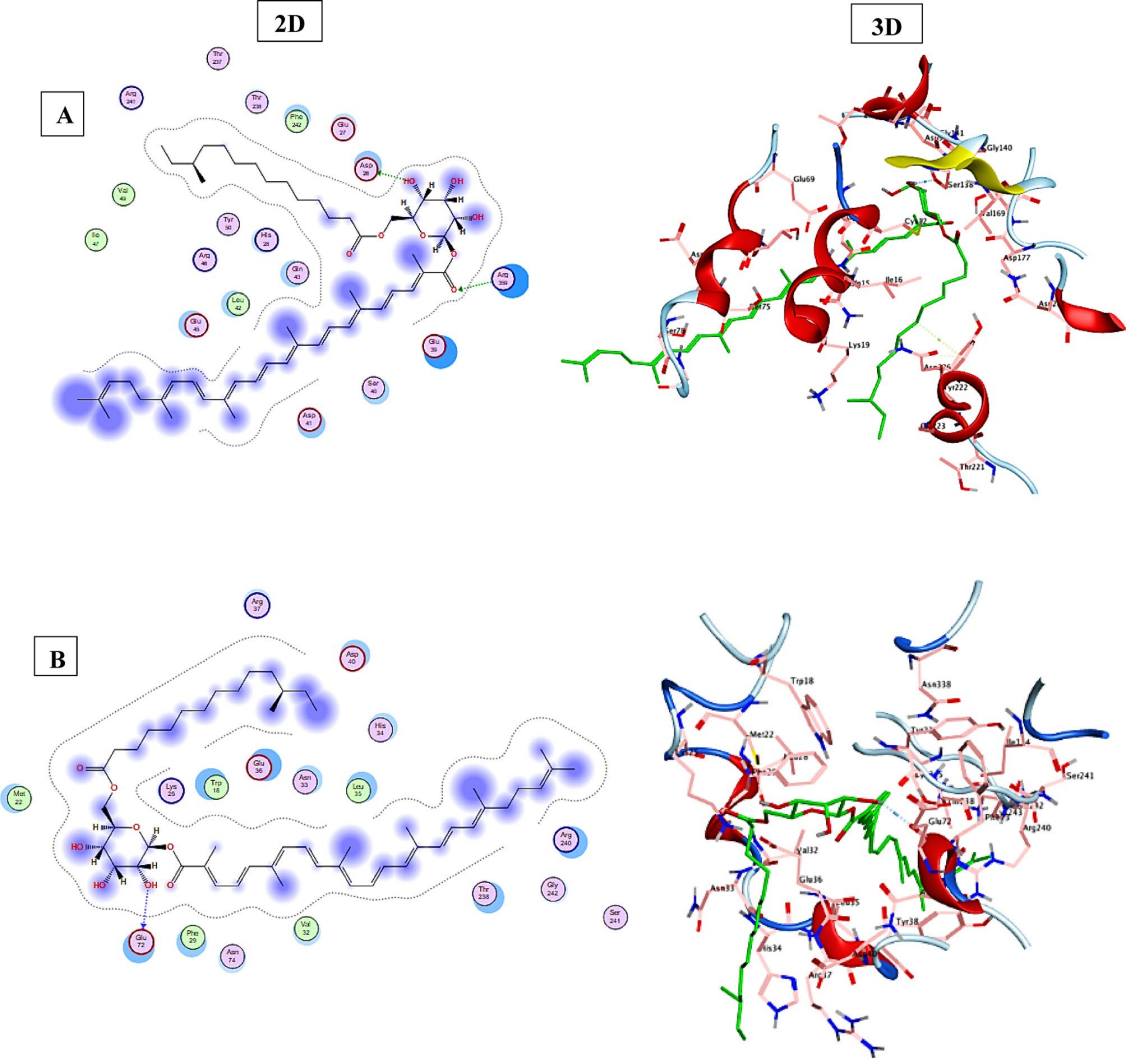

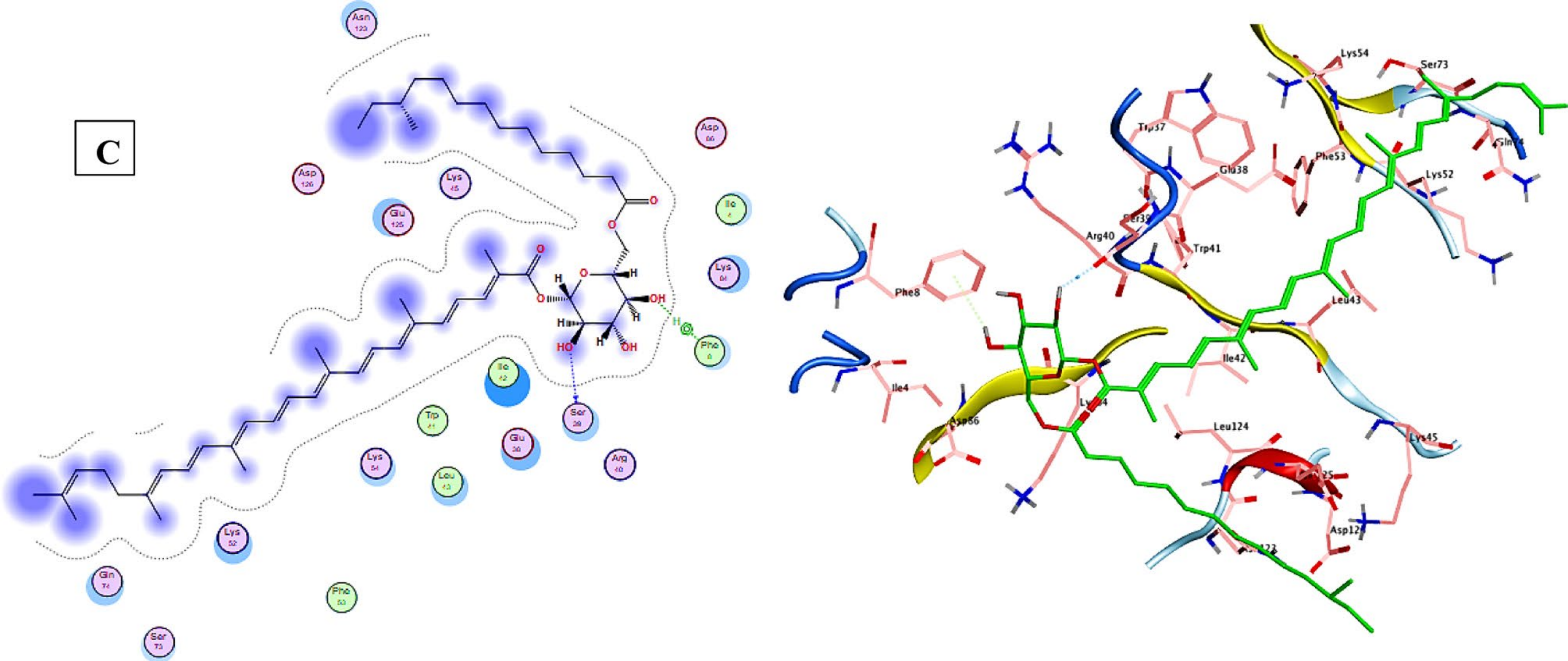
Results of molecular docking illustrating the 2D and 3D binding interactions for STX residue docked and minimized in the binding pocket of *T. spiralis* target proteins. (**A**) Ts-β-tubulin, (**B**) cathepsin F (Ts-CF1), and (**C**) calreticulin (Ts-CRT).

Employing the “molecular operating environment (MOE) version 2019.0102,” a molecular docking study was carried out to enhance the comprehension of the binding interactions of the STX residue and the *T. spiralis* target proteins crucial to the parasite’s life cycle, as shown in Fig. 10. The docking results of STX into the binding pocket of the co-crystallized β-tubulin structure demonstrated that STX exhibited a binding score of −8.7283 kcal/mol, compared to ABZ (score= −6.9504 kcal/mol). This interaction is characterized by hydrogen bonding with Ser138, alongside two hydrophobic interactions with Tyr222. The interactions between the STX and the homology-modeled 3D protein structures, Ts-CRT and Ts-CF1, were further examined through docking analysis. The results of Ts-CF1 docking revealed that STX displayed a hydrogen bond interaction with Glu232, with a binding score of −10.603 kcal/mol compared to ABZ (score= −6.1692 kcal/mol). In relation to Ts-CRT, the docking results for STX indicated a binding score of −8.1168 kcal/mol in in contrast to ABZ (score= −5.7630 kcal/mol), demonstrating a hydrogen bonding interaction with Ser39, alongside a hydrophobic interaction with Phe8. Consequently, it can be inferred that STX may impede the activity of critical proteins in *T. spiralis*.

## Discussion

The advanced development of therapeutic drugs necessitates an in-depth comprehension of pharmacokinetic and toxic properties. The prediction of this data greatly improves the possibility of success while simultaneously decreasing the time needed for drug development^35^. ADMET predictions are commonly employed to assess the characteristics and suitability of a compound for pharmaceutical use^19,36^. In this study, the computed models of STX showed positive pharmacokinetic features of STX, including a negative effect on metabolic rate, biodegradability in the environment, and a low probability of being toxic or mutagenic, compared to ABZ. However, inadequate absorption and permeability decrease the oral bioavailability of STX. Therefore, a promising drug delivery carrier was formulated to overcome this drawback and enhance the therapeutic ability of STX as a promising antiparasitic agent.

This was accomplished through the encapsulation of STX within niosomes, which are cholesterol-based vesicular carriers created using non-ionic surfactant. The encapsulation of hydrophilic and lipophilic medicines is possible with niosomes^37^. When compared to other vesicular carriers, they offer numerous benefits. These include a higher degree of physicochemical stability and a lower cost in comparison to liposomes, the original vesicular carrier prototype^38^. Niosomes demonstrated encouraging potential for increased biological activity in the defense against pathogens^21^. It was hypothesized that these carriers could increase the drug’s permeability through the parasite membrane, have a greater inhibitory effect on the parasite, and have fewer harmful effects on healthy cells^39,40^. Our decision to use niosomes as the delivery mechanism was supported by these data. In this investigation, cholesterol was principally administered using niosomes manufactured with span 60 as the primary surfactant. The incorporation of cholesterol as a membrane stabilizing agent has the potential to improve the drug retention and entrapment^41^. As anticipated, the prepared vesicles exhibited a spherical form. The observed dimensions of the vesicles ranged from 100 to 200 nm in the nanoscale spectrum. There is a consensus with the morphology and size values of the reported data^42,43^. It is critical to highlight that the characteristics of the drug and the specific type of lipid are pivotal determinants of the efficiency of drug entrapment, particularly for lipophilic drugs^24^. Considering the lipophilic nature of STX, it is clear that the STX predominantly localized in the vesicle’s lipid core, thereby emphasizing the recorded drug loading and entrapment efficiency. In light of the values reported by other researchers, the observed entrapment efficiency falls within the appropriate range^20,44,45^.

To the best of our knowledge, this is the inaugural report on the influence of STX in the context of combating trichinellosis. This entailed an examination of the impact of free and niosomes-encapsulated STX on various stages of *T. spiralis*. First, the therapeutic efficacy of STX as an antiparasitic agent was determined in vitro. The findings of this investigation demonstrated that niosomal encapsulation of STX significantly increased the death rate of muscle larvae compared to free STX. This observed result can be ascribed to the vesicles’ instigation of ultra morphological changes. It is conceivable that the enhanced membrane permeability of the noisome is responsible for the changes in parasite morphology^46^. In addition to mortality, muscle larvae experienced notable alterations in their cell walls, including membrane destructions and surface blebs, as evidenced by electron microscopy. Cuticle integrity, a crucial component of body wall of *Trichinella*, along with the somatic musculature and hypodermis, is required for osmoregulation and determines the parasite’s shape, sustenance, and defense^30,47^. Thompson and Geary discovered that transcuticular passive diffusion serves as the primary mechanism for drug entry into helminths, leading to the development of anti-anthelmintics designed to compromise the integrity of the worm’s surface^48^. Blebbing transpires as the parasite endeavors to restore the compromised surface membrane in reaction to pharmacological intervention. Consequently, tegumental alterations may serve as a reliable signal of a drug’s potential anthelmintic efficacy^49^.

Anthelmintic agents can inflict damage upon their cuticle or cytoskeleton or impair parasite metabolism, ultimately resulting in paralysis and subsequent mortality^50^. Hence, the effective antiparasitic activity of STX can primarily be ascribed to its chemical nature of carotenoids. Briefly, the biological potential of carotenoids is primarily attributed to the generation of reactive oxygen species, which in turn causes oxidative stress and alterations in cell permeability^18^. Garcia-Bustos et al. postulated that the pro-oxidative traits enhance the anthelmintic potential by interfering with the helminth parasites’ decoupling oxidative phosphorylation, energy generation, and attaching to the glycoprotein on the parasite’s cuticle, ultimately leading to their demise^51^. Furthermore, Coronel et al., demonstrated that carotenoids have the potential to uncouple specific reductase-mediated mechanisms that impede helminth parasites’ energy production capabilities^52^. A plethora of research studies have proven the encouraging potential of carotenoids in the management of parasitic infections^14,53,54^. Furthermore, the antiparasitic activity was enhanced through the niosomal encapsulation of STX, as demonstrated by the eradication of larval counts. Numerous research studies clarifying the mechanism behind increased efficacy after encapsulation within niosomes as vesicular carriers have focused on the propensity of vesicular systems to adsorb to the pathogen cell membrane^21,55^. Other reports have proposed the fusion of vesicles with the cellular surface. An increased influx of medication into the microorganisms can be facilitated by the adsorption or fusion process, which can render the cell membrane more permeable^56^. The extent of the antiparasitic activity enhancement that is consistent with nano-based formulation is supported by the documented findings observed across an array of parasites^57,58^. Hassan et al. reported that the nano-formulation of hydrazones, compared to the synthesized hydrazone, exhibited exceptional activity against Toxoplasma infection, resulting in a substantial reduction in the burden of brain cysts^57^. Another example is the chitosan-coated nanocarrier, which improved the efficacy of miltefosine in combating *T. spiralis* infection in mice across different life cycle stages, including the intestinal, migratory, and muscular phases, in contrast to its free form^58^. However, to date, there is no published data reporting the antiparasitic potential of STX in drug solution or niosomal dispersion.

In light of the enhanced impact of niosomal encapsulation of STX, the study expanded to assess its in vivo efficacy. The in vivo evaluation entailed the oral delivery of the drug, either in solution form or as niosomes, thereby simulating the authentic administration process in humans. In the current study, the obtained results revealed the superior activity of STX-niosomes in the eradication of adult worms and diminishing the larval count compared to the drug solution and reference drugs. It is posited that vesicular systems may either adhere to or merge with the organism’s surface, leading to the direct conveyance of a substantial concentration of the drug into the organism, emphasizing the augmented activity of niosomal encapsulation^46^. Niosomes have proven this impact in their anti-trichinellosis and other anti-parasitic trials^39,58^. Moreover, the histological deterioration observed in the small intestine and skeletal muscles of infected mice was mitigated after treatment. An important barrier against gastrointestinal nematode infections is the mucus layer, a dynamically produced matrix that is carefully controlled^31^. Hyperplasia of goblet cells and increased mucin synthesis, which is associated with parasite expulsion, are symptoms of a *T. spiralis* infection, as many authors have before described^31,59^. Groups receiving combination therapy demonstrated the most significant enhancement in reducing goblet hyperplasia, focal peri-capsular plasma-lymphocytic inflammatory cellular infiltration, exhibiting the fewest trichina capsules with degeneration, and reinstating normal architecture. These findings corroborate those of a prior study that found that niosomes reduced *T. spiralis* infection burden and inflammatory expression surrounding trichina capsules^60,61^.

Monitoring the life cycle of *T. spiralis* demonstrated that β-tubulin, which is implicated in microtubule polymerization, and cathepsin F specific (Ts-CF1), a cysteine protease found in the stichosome and cuticle, are crucial for its life stages, thereby supporting their potential as therapeutic targets for *T. spiralis* infection^8,62^. Moreover, *T. spiralis* secretes calreticulin protein (Ts-CRT) at various stages of muscle larvae and adult worm development; this protein disrupts, interacts with, and overlaps with the host immune system, allowing *T. spiralis* to evade both the innate and adaptive immune systems of the host. Therefore, targeting this protein receptor could be an effective strategy to combat *T. spiralis* infection^63^. To reveal the manner in which STX inhibits *T. spiralis* infection, the 3D structures of Ts-CF1 and Ts-CRT were prepared using a homology modeling protocol. Based on our molecular docking results, STX may have the capacity to influence the target enzyme systems in a manner that is comparable to or superior to that of the reference drug, indicating their possible antiparasitic efficacy against *T. spiralis* at various stages of its life cycle.

## Conclusion

The oral administration of STX via niosomal nanocarriers presents a promising delivery approach for the treatment of trichinellosis. These vesicles have the potential to augment the efficacy of STX in both in vitro and in vivo settings against *T. spiralis*. The enhanced oral bioavailability, along with the capacity of niosomal dispersion to induce ultrastuctural tegumental alterations resulting in paralysis and subsequent mortality, contributes significantly to the heightened activity observed. Moreover, the results of our molecular docking analysis indicate that STX may induce larval mortality through its capacity to disrupt enzymatic pathways essential to the life cycles of *T. spiralis*. The observed efficacy of STX-loaded niosomes paves the path for potential application and translation into clinical practice. However, further studies are required to assess the pharmacokinetic properties of STX.

## Supplementary Information

Below is the link to the electronic supplementary material.

Supplementary Material 1

## Data Availability

All data generated or analyzed during this study are included in this published article.
