# Supplementary material for: Staphyloxanthin loaded niosomal nanocarrier augments its anthelmintic activity against Trichinella spiralis infection in mice
Source: Sci Rep. 2025 Sep 12;15:32446. doi: 10.1038/s41598-025-17936-9 (PMC12432262; doi:10.1038/s41598-025-17936-9)
Supplement: Supplementary file 1 — Supplementary Material 1 [file 41598_2025_17936_MOESM1_ESM.docx]

**A**


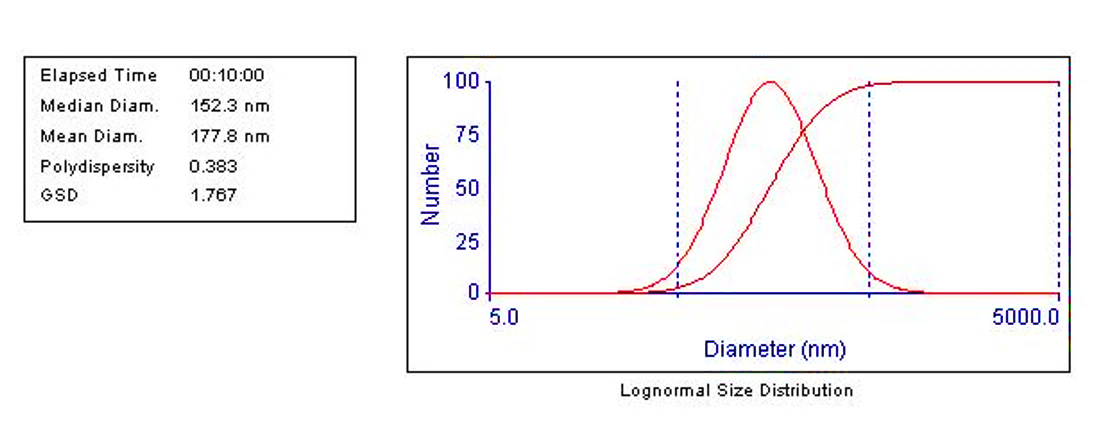


**B**


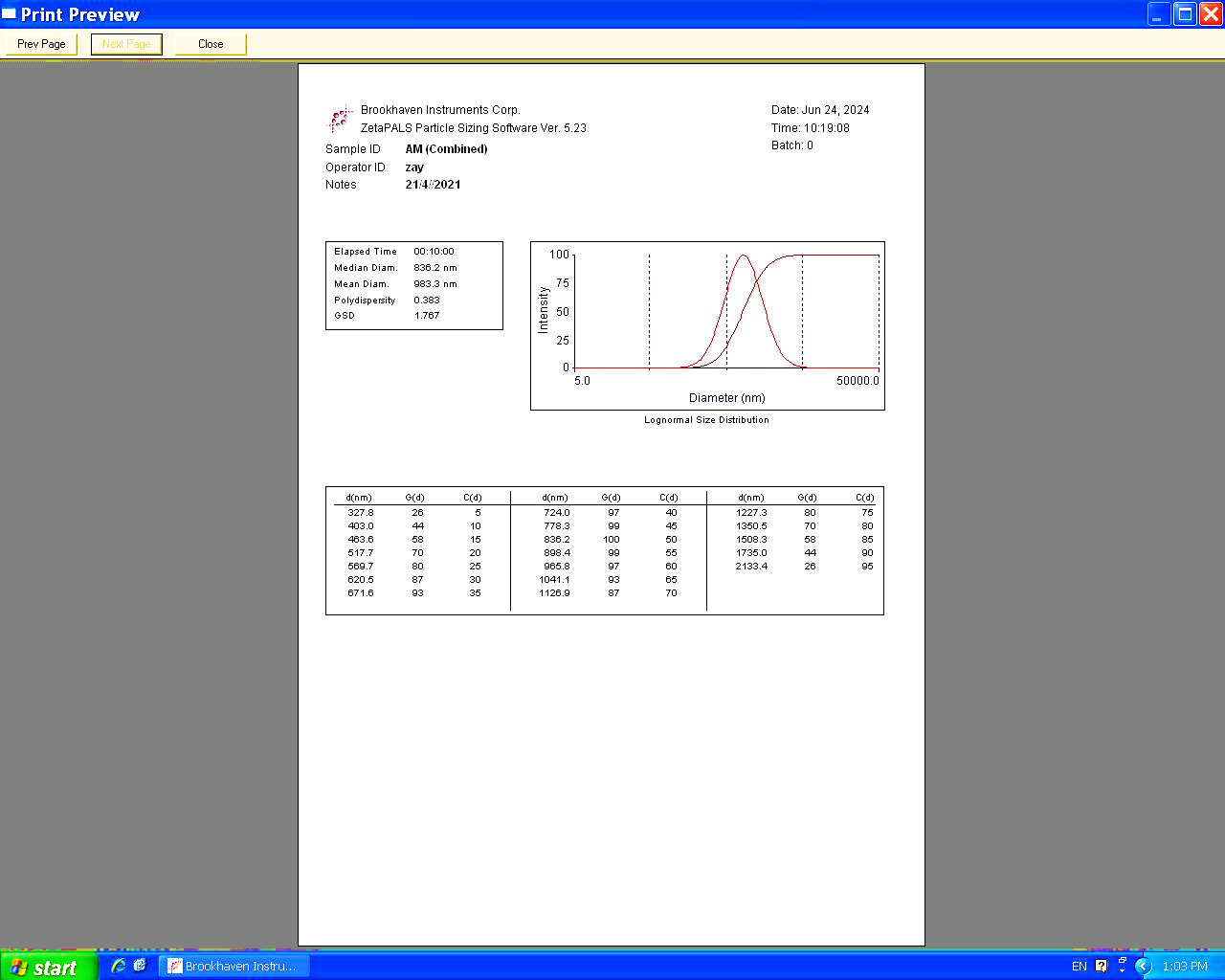


**Figure S1** Stability of the formulated niosomes over a 30-day period at different temperatures of 4 °C and 25 °C. (**A**) DLS analysis showed niosomal size in the nanoscale range in case of refrigeration. (**B**) DLS analysis showed aggregation of nanovesicle after storge at room temperature.
